# Supplementary figures and images for: A single-cell atlas of the sexually dimorphic Drosophila foreleg and its sensory organs during development
Source: PLoS Biol. 2023 Jun 28;21(6):e3002148. doi: 10.1371/journal.pbio.3002148 (PMC10335707; doi:10.1371/journal.pbio.3002148)

**A****24h**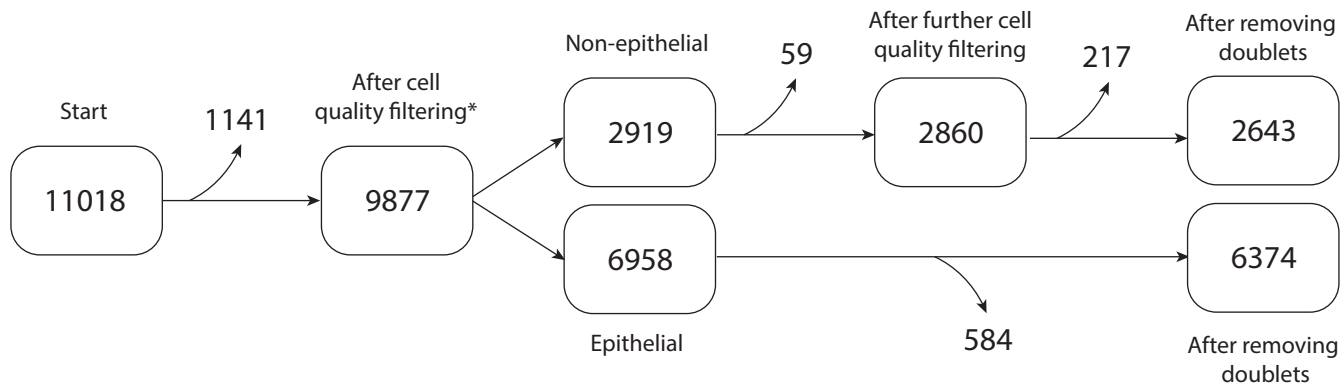**30h**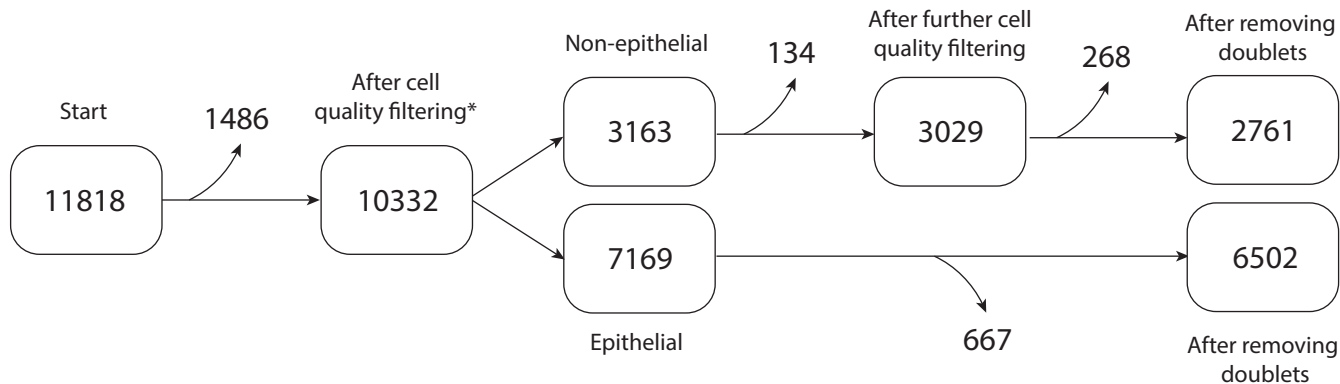**B**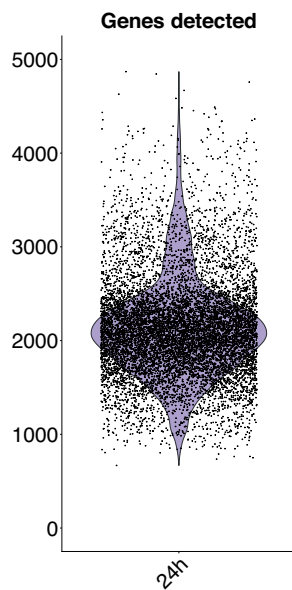**C**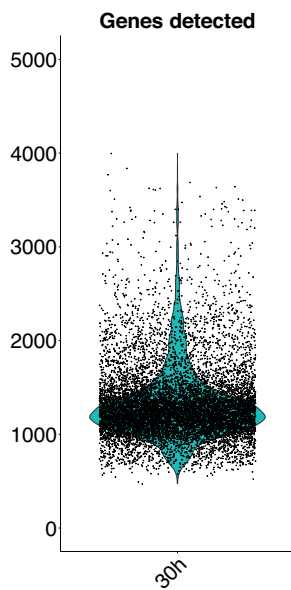**D**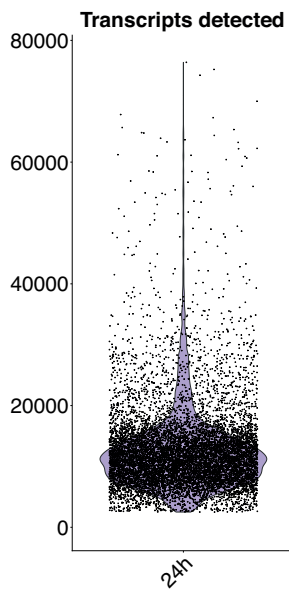**E**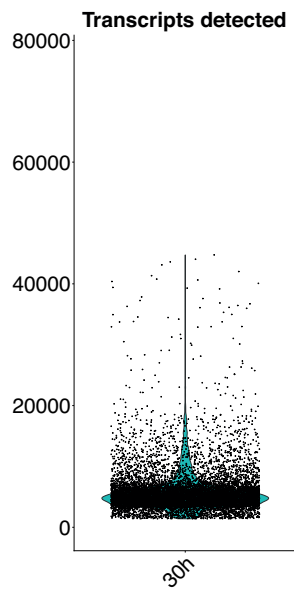

Supplement: S1 Fig — (A) A schematic detailing how many cells were filtered out at each stage of processing. Cells were initially filtered from the full dataset and retained based on the number of genes detected per cell (24 h: >450 and <5,000; 30 h: >425 and <5,000), transcripts detected per cell (24 h: >2,500; 30 h: >1,400), and the percentage of transcripts that map to mitochondrial genes (24 h and 30 h: <10%). The datasets were then split into epithelial and nonepithelial cells based on cluster identity (the asterisks here relate to panels (B-E)). Additional filtering was then performed on the nonepithelial cells, removing cells with >5% mitochondrial reads and nonsheath bristle cells in which >2 transcripts of the sheath marker nompA were detected, which likely correspond to undissociated doublets. Further doublets were then identified in each dataset using DoubletFinder [181] and removed. In the 30 h dataset, an additional 9 cells positive for the hemocyte marker NimC4 were identified at the interface between the mechanosensory socket and shaft cluster and at the edge of the bract cluster. These putative hemocyte–bristle cell doublets were also removed. (B-E) Violin plots showing the distribution of (B) genes detected per cell in the 24 h dataset, (C) genes detected per cell in the 30 h dataset, (D) transcripts detected per cell in the 24 h dataset, and (E) transcripts detected per cell in the 30 h dataset. Panels show the distribution in the full datasets after initial filtering based on cell-level quality control metrics (i.e., the distributions at the positions indicated by asterisks in (A). Numerical data with cell barcodes are listed in S3 Data (24 h) and S4 Data (30 h). Code for generating the figure is available at https://www.osf.io/ba8tf. (PDF) [file pbio.3002148.s001.pdf]

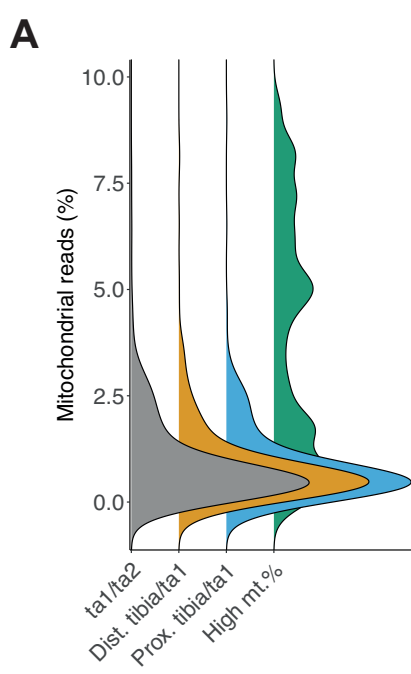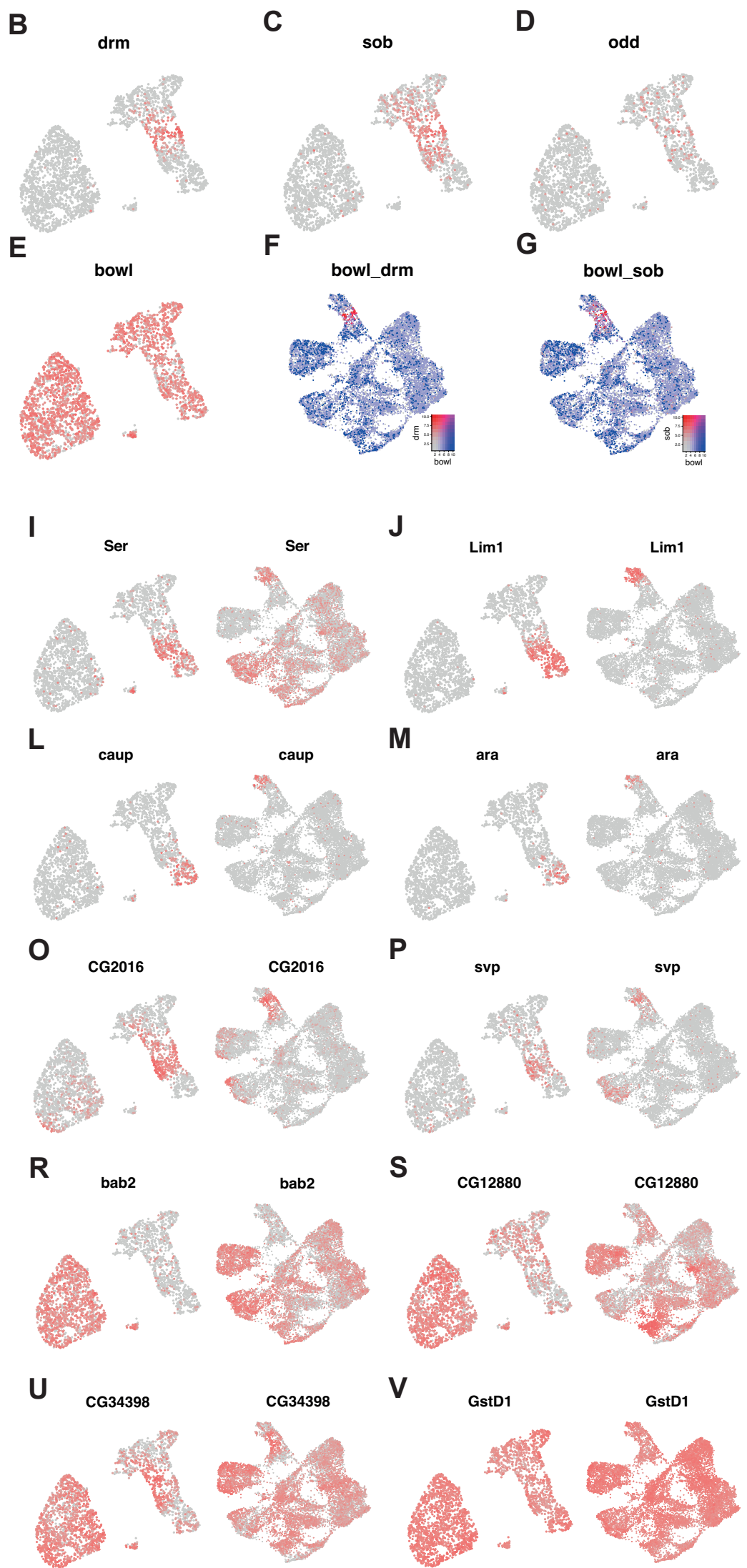

Supplement: S2 Fig — (A) The smallest of the clusters identified in our clustering analysis (Fig 3F) showed highly variable internal expression patterns (i.e., the top markers of this cluster were generally expressed in a relatively small number of its constituent cells). Moreover, this cluster, colored dark green in this plot, showed a markedly higher representation of cells with high mitochondrial read counts, suggesting it may be composed of or enriched for damaged cells. We therefore excluded it from further analysis. Numerical data with cell barcodes are listed in S5 Data. (B-E) UMAP plots of the joint dataset overlaid with the expression of odd-skipped family transcription factors. drm, sob, and odd are known to be expressed in the distal edge of each leg segment except tarsal segments 1–4 [52]. Consistent with its widespread expression among epithelial cells in our dataset, bowl has been shown to display an overlapping but broader expression pattern (extending into tarsal segments 1–4) than odd, drm, and sob [52]. (F, G) UMAP plots of the full joint and nonjoint epithelial dataset overlaid with the expression of bowl (blue) and drm (red; F) or sob (red; G). (H-V) For each panel, a UMAP plot of the joint dataset (left) and full joint and nonjoint epithelial dataset (right) is overlaid with the expression of a given gene identified during the joint differential gene expression analysis. (H, I) CG1648 and Ser show widespread expression among the proximal tibia/ta1 joint and epithelial cells but are excluded from the other joint clusters. This is expected for Ser as Ser+ cells form patterning boundaries in the developing leg that activate joint formation in distally adjacent cells via Notch [193]. (J-N) Lim1, trh, caup, ara, and pdm2 show specific expression in the proximal tibia/ta1 cluster. Of these, Lim1 is known to be expressed in the tibia where it is required for specification of the tarsus ([194]; see also Fig 5V); nub1 alleles give rise to compromised leg development [195]; p [file pbio.3002148.s002.pdf]

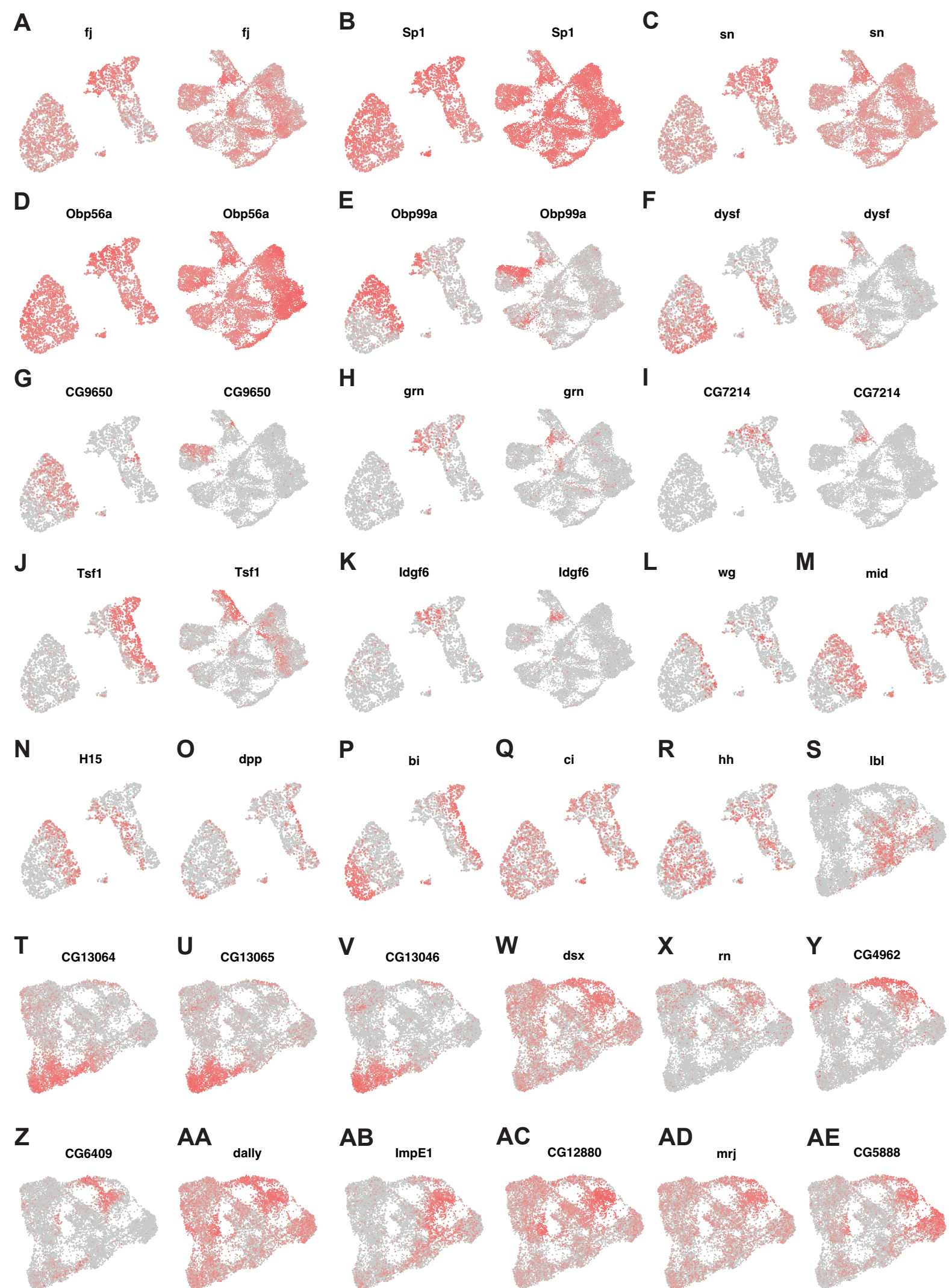

Supplement: S3 Fig — (A-K) For each panel, a UMAP plot of the joint dataset (left) and full joint and nonjoint epithelial dataset (right) is overlaid with the expression of a given gene identified during the joint cluster differential gene expression analysis. (A-D) Several of the top differentially expressed genes (DEGs) for the distal tibia/ta1 (fj, Sp1, sn, Obp56a) do not appear to be specific, rather they show localized enrichment in joint cells alongside widespread expression in nonjoint epithelial cells. One such gene is fj, which we find widely expressed across all joint and nonjoint clusters but enriched in the distal tibia/ta1 cluster (A; Fig 3I). fj is known to be required for regional growth along the leg’s proximal–distal axis and in imaginal discs shows rings of expression that are complementary to Nub [54,197]. However, our data show a separation between the regions of peak nub and fj expression (compare Fig 3I with Fig 3D). Several of the remaining top DEGs for the ta1/ta2 (E-G) and distal tibia/ta1 (H-K) joint clusters show more specific expression patterns. (L-R) UMAP plots of the joint dataset overlaid with the expression of genes specifying positional identity. Positional identity is clear at the level of the dorsal–ventral axis (ventral: wg, mid, H15; dorsal: dpp, bi; L-P), but not anterior–posterior axis (anterior: ci; posterior: hh; Q, R). (S-AE) UMAP plots of the nonjoint epithelial dataset overlaid with the expression of genes identified as enriched within subregions. Data and code for generating the figure are available at https://www.osf.io/ba8tf. (PDF) [file pbio.3002148.s003.pdf]

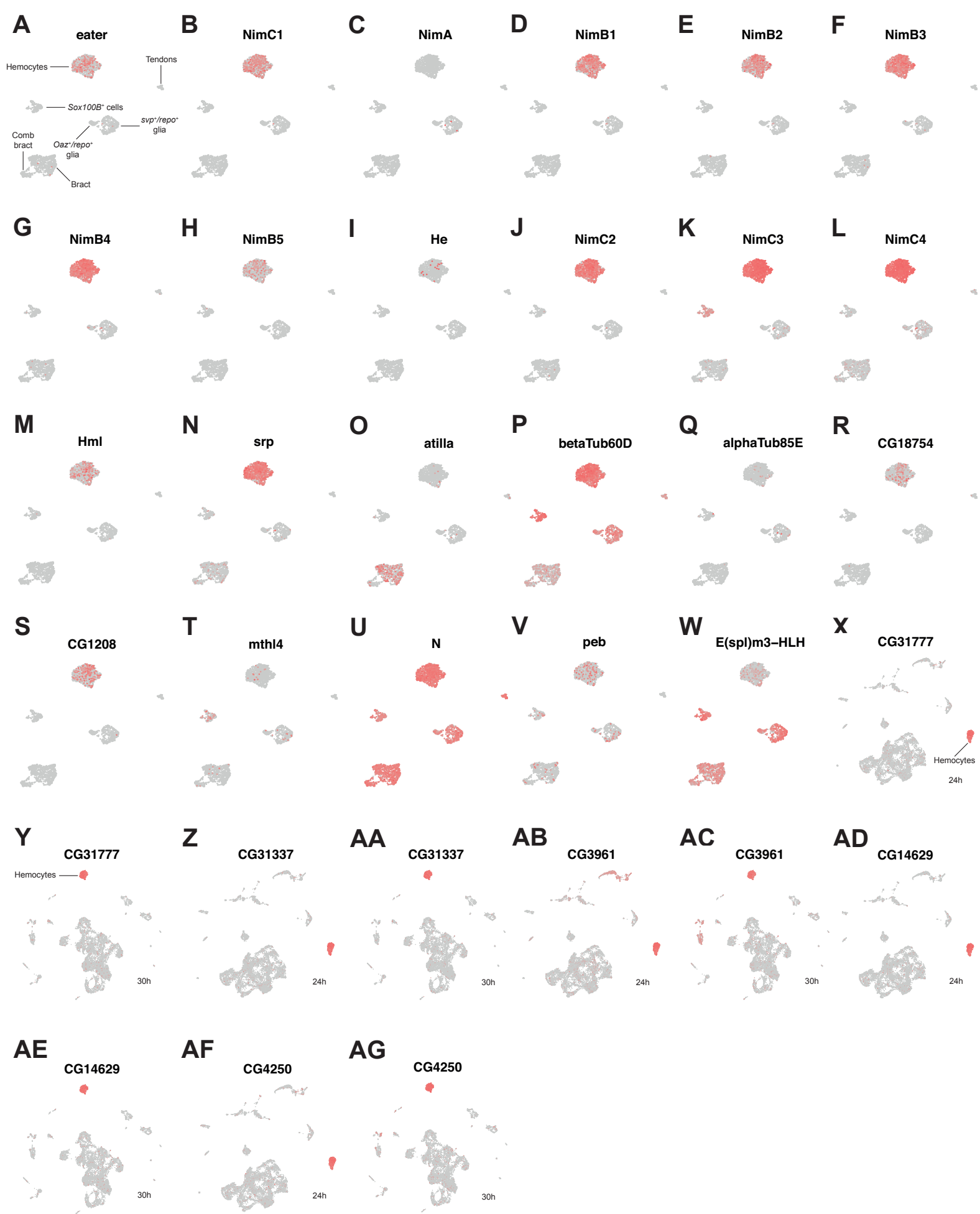

Supplement: S4 Fig — (A-W) UMAP plots of the nonsensory dataset overlaid with gene expression. (A-N) A selection of hemocyte markers, most of which are part of a cluster of NIM-repeat containing genes on chromosome 2 that also includes the hemocyte-specific He. RT-PCR work has previously shown that all of these, except nimA, are transcribed in larval hemocytes [69]. We find that all these NIM genes, except nimA, are expressed in our hemocyte cluster, as are Hml and srp, which are known to be expressed in both differentiating and mature plasmatocytes [67,68]. (O-T) Genes identified by Tattikota and colleagues ([70]; see also [72]) as enriched in lamellocytes. We saw no obvious subclustering in relation to these genes: They were either widely expressed among hemocytes (betaTub60D, CG1208), too patchily expressed among hemocytes to reflect a clear subpopulation (atilla, alphaTub85E, CG18754, mthl4), or absent from our dataset entirely (CG31219, CG14610, CG15347, CG12133). (U-W) The same study found that crystal cells showed highest enrichment of PPO1, PPO2, lz, N, peb, and E(spl)m3-HLH. As with lamellocytes, we saw no obvious subclustering in relation to these genes: PPO1, PPO2, and lz were absent from our dataset, and N, peb, and E(spl)m3-HLH showed nonspecific expression. We also mapped the top markers of many of the plasmatocyte subclusters identified by Tattikota and colleagues (Mmp1, IM18, CecA2, CecC, Mtk, DptB, Drs, Prx2540-1, Prx2540-2, CG12896, Abl, Snoo, CG15550, CG6023, mthl7, Cys, CG8860, and COX8), but saw no clear subclustering. (X-AG) Alternating UMAP plots of the full 24 h (X, Z, AB, AD, AF) and 30 h (Y, AA, AC, AE, AG) datasets overlaid with expression of a subset of hemocyte marker genes identified in this study. Data and code for generating the figure are available at https://www.osf.io/ba8tf. (PDF) [file pbio.3002148.s004.pdf]

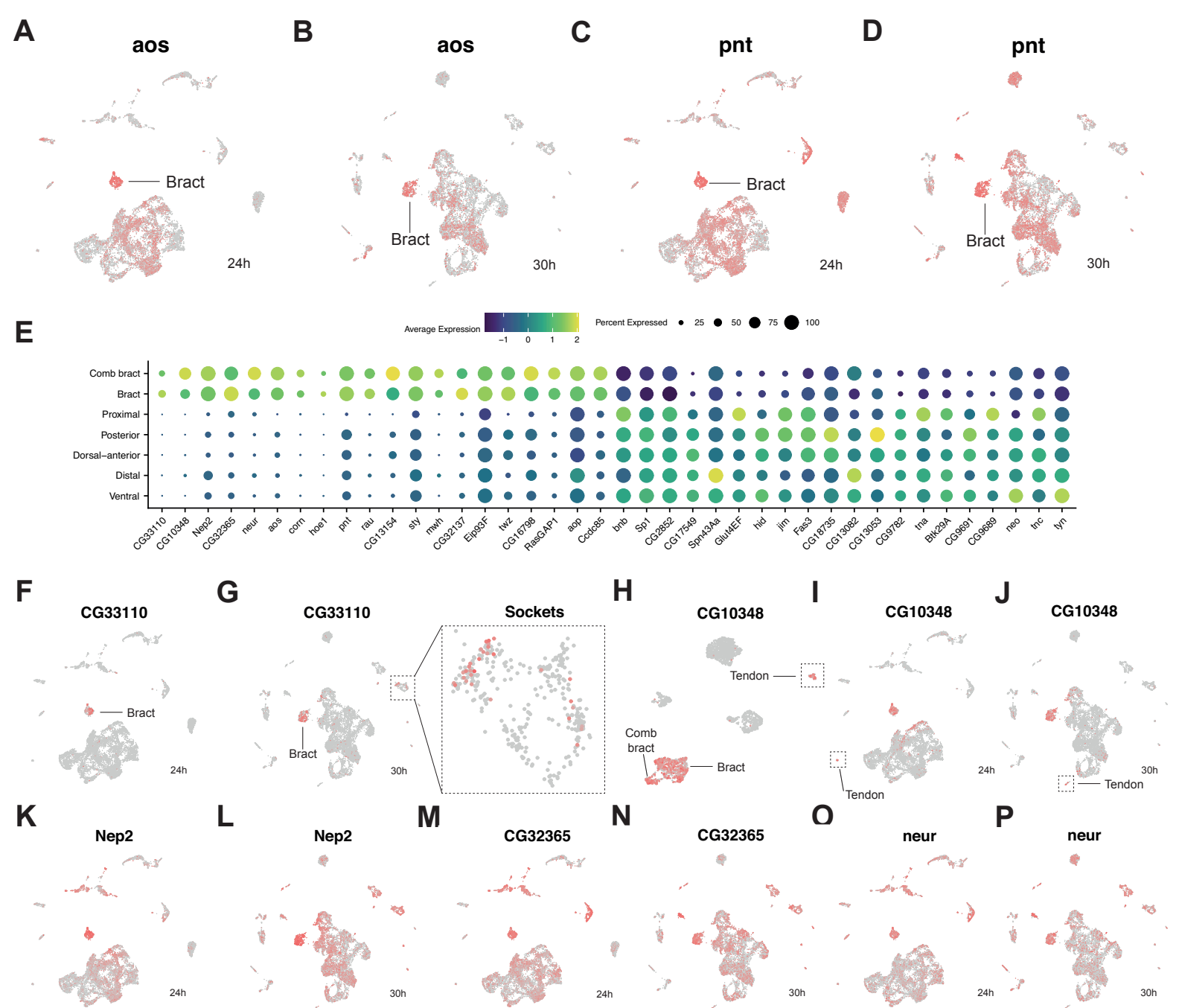

Supplement: S5 Fig — (A-D) Alternating UMAP plots of the full 24 h (A, C) and 30 h (B, D) datasets overlaid with expression of the bract markers pnt and aos, EGFR signaling components with known roles in bract formation [74–76]. (E) A dot plot of the top 20 differentially expressed genes from a bract versus nonjoint epithelial comparison. Although the comparison was made between all bracts and all nonjoint epithelial cells, the breakdown per subcluster is depicted (“distal” here refers to the sex comb bearing region). Of these, the ecdysone-induced transcription factor Eip93F (also called E93), which we found to be up-regulated in bract cells, is known to be expressed in the epithelial cell that will develop as a bract, where it enables Dll to respond to EGFR signaling [76]. (F) UMAP plot of the full 24 h dataset overlaid with the expression of the top bract marker, CG33110, which encodes a predicted fatty acid elongase. (G) As (F) but in the 30 h dataset, alongside an inset zooming in on the socket cell cluster. The expression of CG33110 in sockets was more pronounced in the 30 h compared to 24 h dataset. (H) A UMAP plot of the nonsensory dataset overlaid with expression of CG10348, a top bract marker. Expression is clearly enriched in bract cells, as well as tendon cells, which are highlighted by a dashed box. (I-P) Alternating UMAP plots of the full 24 h (I, K, M, O) and 30 h (J, L, N, P) datasets overlaid with several top bract markers. In the case of most of these genes, expression is detected outside the bracts and often enriched in the sensory organ cells. Data and code for generating the figure are available at https://www.osf.io/ba8tf. (PDF) [file pbio.3002148.s005.pdf]

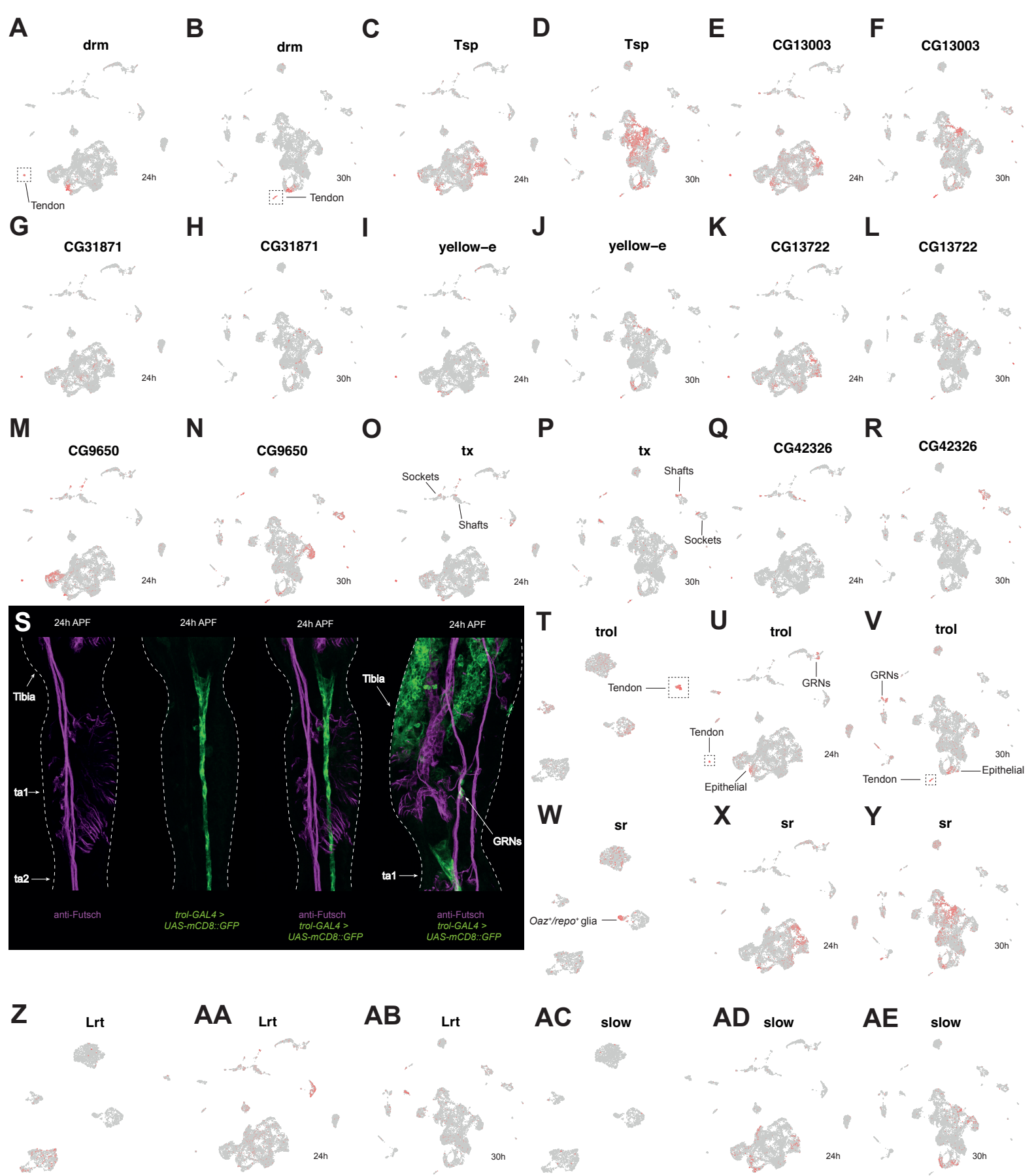

Supplement: S6 Fig — (A-R) Alternating UMAP plots of the full 24 h (A, C, E, G, I, K, M, O, Q) and 30 h (B, D, F, H, J, L, N, P, R) datasets overlaid with several top tendon markers. (A-N) For drm, Tsp, CG13003, CG31871, yellow-e, CG13722, and CG9650, expression was observed in both tendon cells and a subregion of epithelial cells. For tx (O, P) and CG42326 (Q, R), we also observed expression in a subset of shaft and socket cells. In both cases, this socket and shaft expression was more widespread at 30 h. (S) Confocal images of 24 h APF male trol-GAL4 > UAS-mCD8::GFP (green) [133] legs counterstained with the neuronal marker anti-Futsch (magenta). The first 3 images show the separate and merged channels from an image of the first tarsal segment. The staining follows much the same pattern showed by anti-Vvl and 1151-GAL4 > UAS-mCherry.nls in the tendon cells (Fig 4G–4J). Note the concentration of staining around the tibia/ta1 joint, the position of the levator and depressor tendons. The right-hand image shows the distal tibia and proximal ta1 with merged channels. Note the presence of extensive trol-GAL4 staining in epithelial cells in the tibia—no equivalent epithelial staining was observed in the tarsus. The epithelial trol-GAL4 staining observed in the tibia was not present in the region proximal to the tibia/ta1 joint. trol-GAL4 staining was also observed in gustatory receptor neurons (GRNs) (see S12 Fig). (T-V) Expression of trol overlaid on the nonsensory (T), full 24 h (U), and full 30 h (V) UMAP plots. Note the expression of trol in a subset of GRNs (as shown in (S) and S12 Fig). Some localized expression is present in a region of the epithelial clusters that corresponds to the proximal tibia/ta1 portion of our joint UMAPs, rather than the distal tibia/ta1 region, and therefore likely reflects the tibia/ta1 joint staining rather than that in the more distal tibia, which falls outside of our dissected region. (W-AE) Alternating UMAPs showing expression of known tendon genes sr, L [file pbio.3002148.s006.pdf]

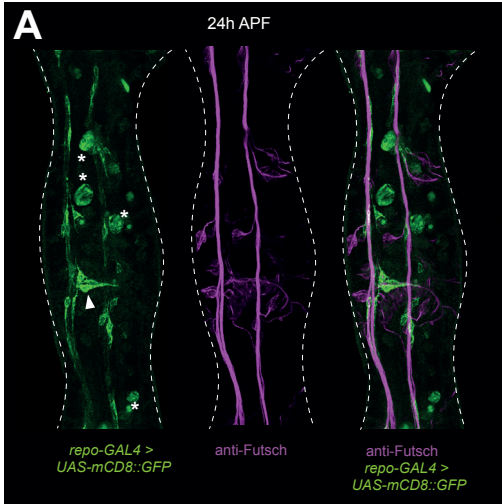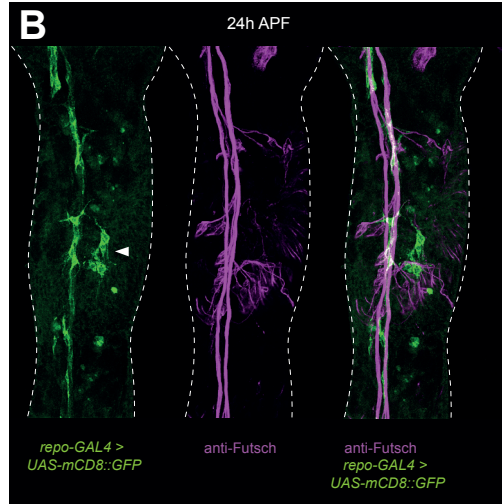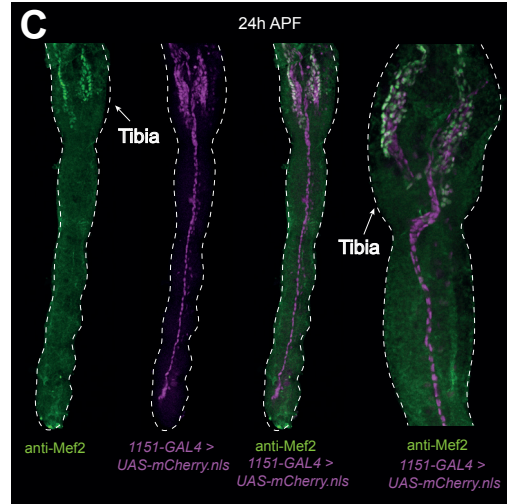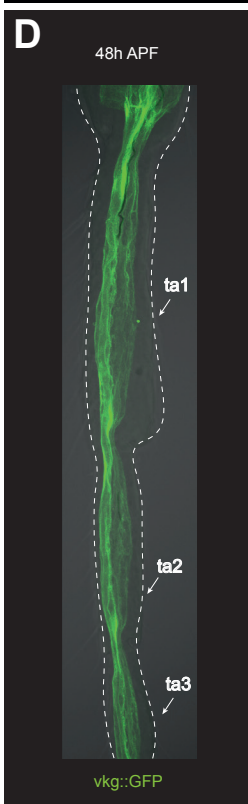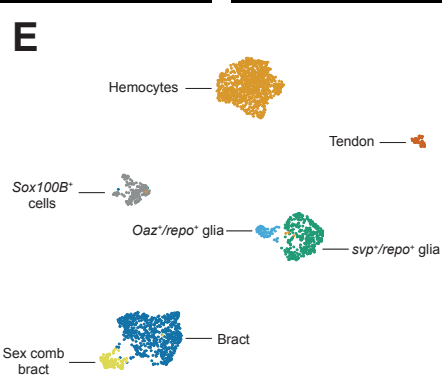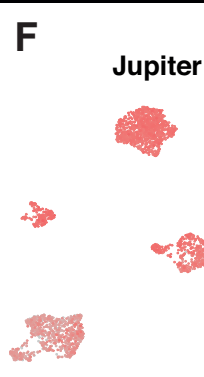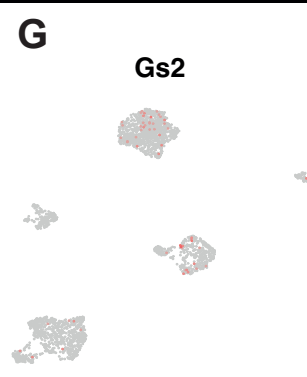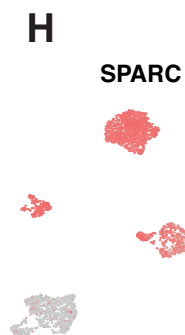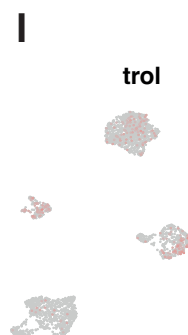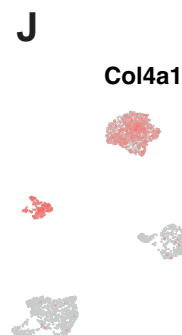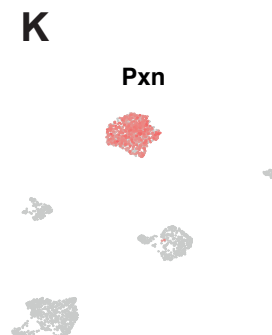

Supplement: S8 Fig — (A, B) 24 h APF male first tarsal segments from repo-GAL4 > UAS-mCD8::GFP (green) counterstained with anti-Futsch (magenta). Note how the staining pattern includes cells hugging the axon trunks, but also, and unlike in the Lim1-GAL4 staining, cells that branch away from them. This latter feature is particularly clear around the sex comb (see white triangles). Note also that some circular, nonspecific fat body staining is present (examples marked by asterisks in (A)). (C) 24 h APF male forelegs from 1151-GAL4 > UAS-mCherry.nls (magenta) males counterstained with anti-Mef2 (green). 1151-GAL4 is a myoblast and tendon cell marker, while Mef2 is a transcription factor essential for cardiac, visceral, and somatic muscle development [203]. Anti-Mef2 staining is restricted to the tibia. (D) Confocal image of a male 48 h pupal leg from a vkg::GFP line. Both transmitted light and GFP channels are shown merged. (E) Annotated UMAP plot of nonsensory cells. (F-K) The UMAP shown in (E) overlaid with the expression of: (F) Jupiter, a perineural glia marker [96] that here shows widespread expression; (G) Gs2, a surface glia marker that here shows patchy expression in the svp+/repo+ cluster [89]. (H-K) A series of extracellular matrix components that show varying expression profiles across repo+ glia, Sox100B+ glia, and hemocytes. Data and code for generating the scRNA-seq elements of this figure are available at https://www.osf.io/ba8tf. (PDF) [file pbio.3002148.s008.pdf]

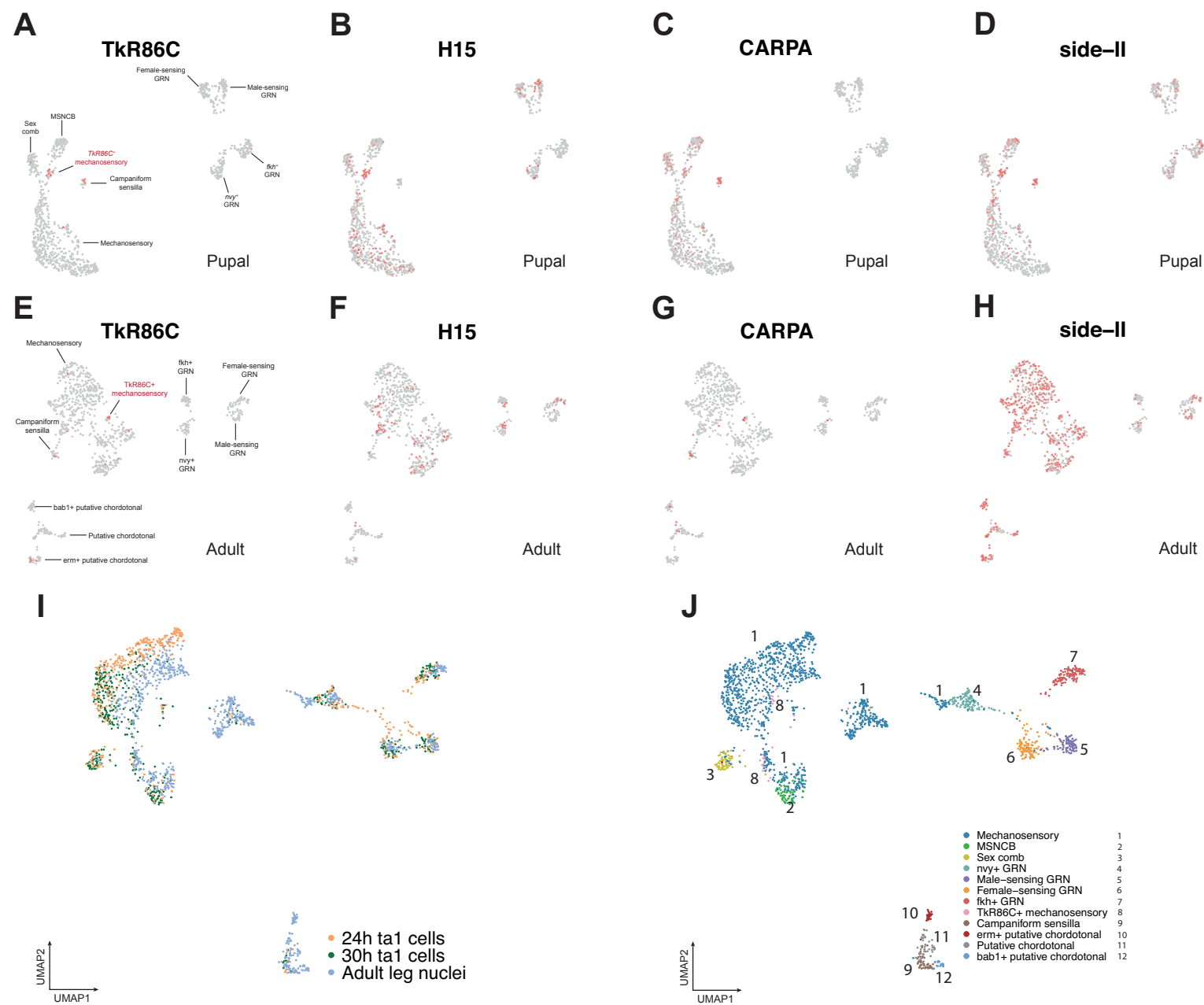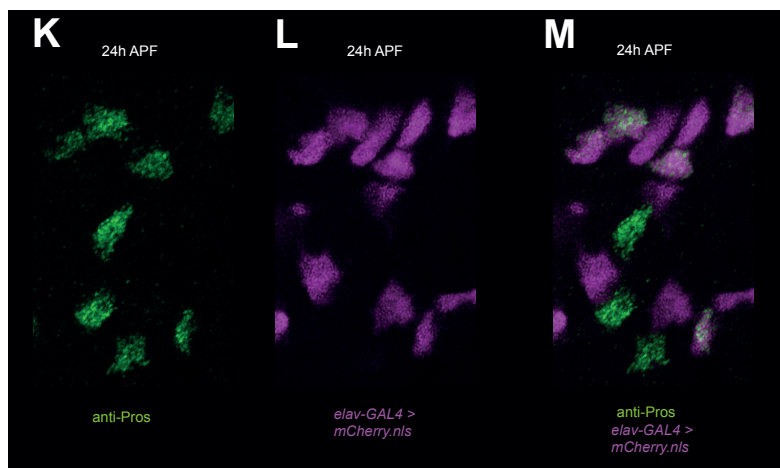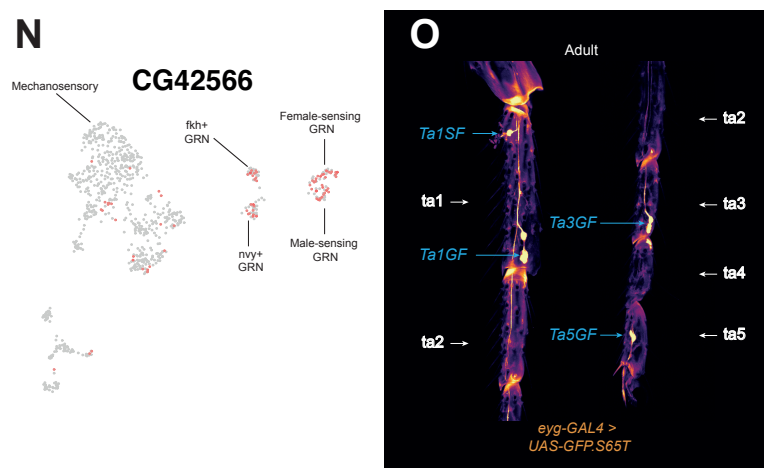

Supplement: S9 Fig — (A-H) In our unsupervised clustering analysis of both the pupal neuron and the FCA neuron datasets, a small subpopulation of mechanosensory neurons clustered separately, labeled in (A) and (E) as TkR86C+ mechanosensory neurons. These clusters were enriched for TkR86C (A, E), which encodes a receptor for the neuropeptide tachykinin and plays a critical role in male-specific neural, circuits that control aggression [198]. (B-D) In the pupal dataset, these cells were also enriched for the ventral marker H15, CARPA, and Side-II. (F-H) However, in the adult data, only CARPA showed any suggestion of being enriched in these cells relative to the other neuron populations. Because of their scarcity, coupled with the absence of strongly specific genes, it’s unclear whether the TkR86C+ cluster represents a distinct population. We cannot rule out that in the pupal data, they may simply correspond to more developmentally advanced mechanosensory neurons and/or a population from a particular subregion of the leg, the clustering of which is driven by the shared expression of positional markers such as H15. (I) A UMAP plot of an integrated dataset of the 24 h APF male first tarsal segment single-cell RNA-seq data, 30 h APF male first tarsal segment single-cell RNA-seq data, and adult all leg male neuron single-nuclei RNA-seq data. Cells are colored according to the dataset of origin. (J) The UMAP plot given in (I) but this time cells are colored according to the cluster annotation they were assigned based on separate clustering and analysis of the pupal cell and adult nuclei datasets (i.e., those presented in Fig 6A and 6G). Note how the nvy+ cluster includes both cells labeled as nvy+ gustatory receptor neurons (GRNs) and mechanosensory neurons. Because of this divergent classification, and the broader differences between the datasets in the tissues, their ages, and the dissociation protocol used, we opted to analyze the 2 datasets separately. (K-M) Confocal images of mechanosensor [file pbio.3002148.s009.pdf]

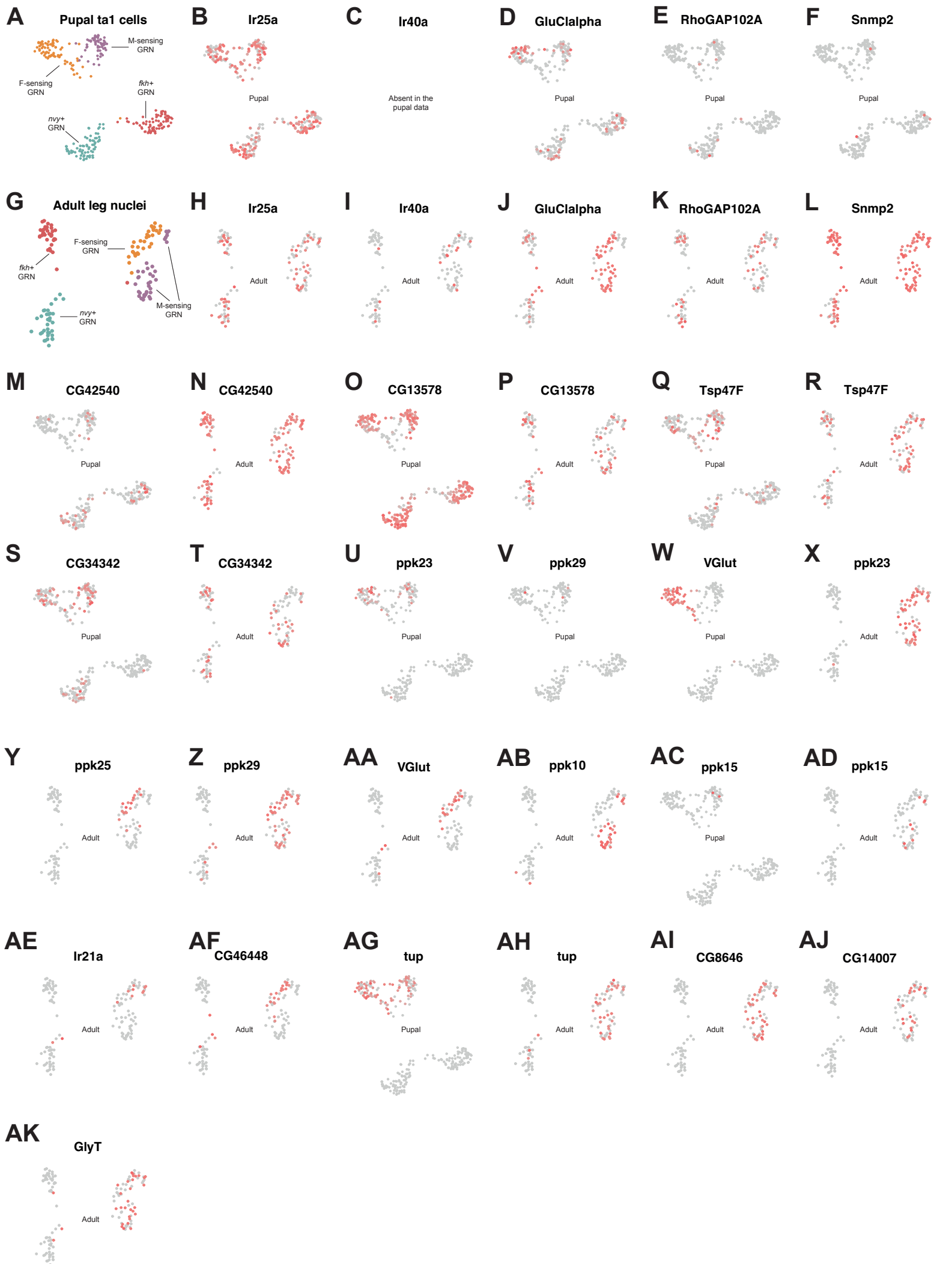

Supplement: S10 Fig — (A-T) UMAPs showing the annotated GRN clusters identified in the integrated pupal neuron data (A) and FCA adult leg data (G) and then overlaid with the expression of genes identified as being specifically expressed or enriched in all GRNs relative to all other neuron populations. (U-AK) The expression of a set of genes that are enriched in the female-sensing and/or male-sensing GRN clusters overlaid on the UMAPs described in (A) and (G). Of these, ppk23 and ppk29 are known from previous work to be expressed in both neuron types, while VGlut is restricted to female-sensing neurons [21,22,123–126]. We additionally detect the transcription factor tup in both populations and ppk10 and ppk15 in the male-sensing population. Note that in (AD), 2 of the 3 ppk15+ cells outside the main body of M-sensing GRN cells fall within the acj6+ region we identified as likely being additional M-sensing cells (see Fig 7AH). Taking the 2 datasets together, ppk15 represents a strong candidate for a gene involved in male pheromone detection, as does ppk10. A gene plotted for just one of the 2 datasets indicates its absence from the other. Data and code for generating this figure are available at https://www.osf.io/ba8tf. (PDF) [file pbio.3002148.s010.pdf]

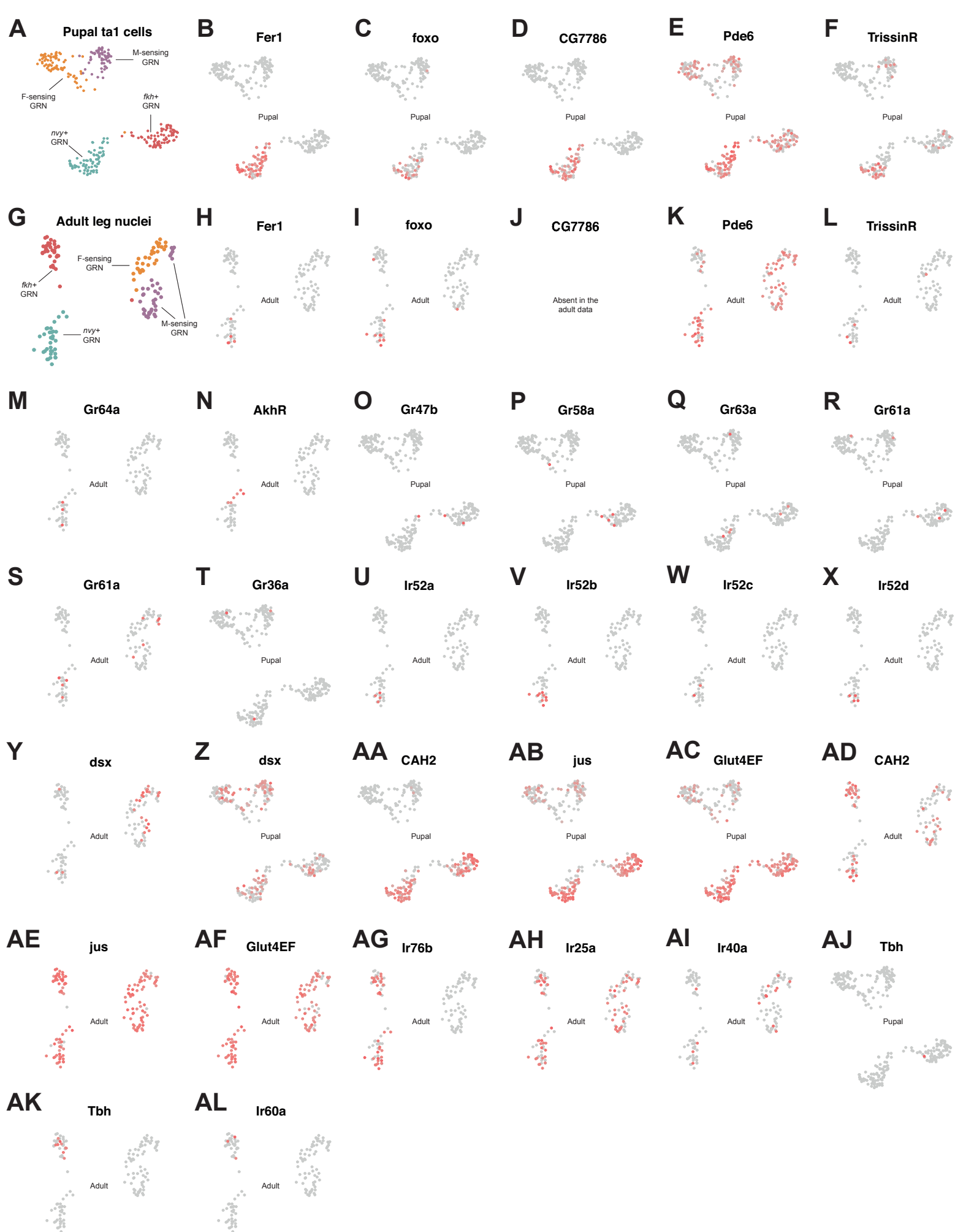

Supplement: S11 Fig — (A-N) UMAPs showing the annotated GRN clusters identified in the integrated pupal neuron data (A) and FCA adult leg data (G) and then overlaid with the expression of genes identified as being specifically expressed or enriched in the nvy+ GRN cluster. (O-T) As well as searching through DEGs, we ran through the expression of all 60 gustatory receptor genes [19,199–202]. Of these, we detected Gr36a, Gr47b, Gr58a, and Gr63a exclusively in the pupal ta1 dataset, Gr64a exclusively in the adult leg dataset, and Gr61a in both. In all cases, expression was limited to just a handful of cells precluding us from confidently assigning Grs to specific GRNs. We failed to detect Gr68a, a receptor that has been shown to be specifically expressed in the male foreleg [3], in either dataset. (U-X) Ir52a-d, the expression of which is shown here overlaid on the UMAP presented in (G), were specifically expressed in the nvy+ GRN cluster and only detected in the adult dataset. (Y-Z) The expression of dsx appears more widespread among the GRNs in the pupal data compared to the adult data. This may reflect differences in the dissected regions, with dsx expression restricted to only the nvy+ and fkh+ GRNs on the foreleg, while dsx is expressed in all fru+ neurons regardless of the leg they’re on. (AA-AL) A selection of genes enriched specifically in fkh+ GRNs or that are shared between fkh+ and nvy+ GRNs. Note how initially restricted expression in the pupal data of CAH2 (AA), jus (AB), and Glut4EF (AF) expands to widespread expression in the adult data (AD-AF). Genes presented for just one of the 2 UMAPs indicates its absence from the other dataset. Data and code for generating this figure are available at https://www.osf.io/ba8tf. (PDF) [file pbio.3002148.s011.pdf]

**A**

24h APF

ta1 →

ta2 →

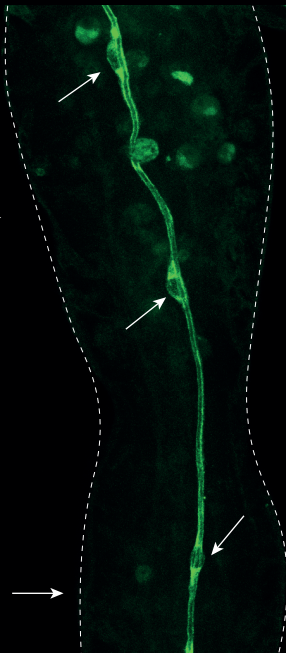

*CG43394-GAL4 >  
UAS-mCD8::GFP*

**B**

24h APF

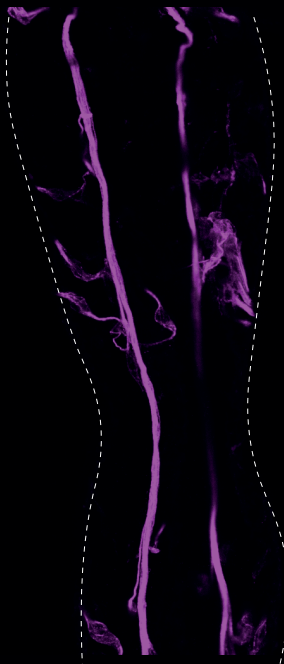

anti-Futsch

**C**

24h APF

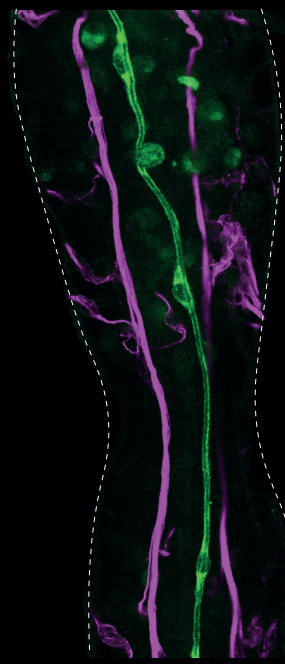

anti-Futsch  
*CG43394-GAL4 >  
UAS-mCD8::GFP*

Supplement: S17 Fig — Confocal images of a 24 h APF male first tarsal segment from CG43394-GAL4 > UAS-mCD8::GFP counterstained with the neuronal marker anti-Futsch. Visible cell bodies are highlighted with arrows in (A). CG43394 was one of the top markers for a cluster we identified as socket cells from chemosensory bristles. This annotation was based on the strong transcriptomic overlap between the cluster and the other socket populations, including the shared expression of the known socket transcription factors Su(H)+ and Sox15+ (Fig 8G). However, the CG43394-GAL4 > UAS-mCD8::GFP staining does not appear to correspond to chemosensory sockets, but rather to a trachea-like channel running through the middle of the tarsal segments. While we cannot exclude the possibility that the cluster we annotated as chemosensory sockets in fact corresponds to these cells, we have reason to doubt that it does. In addition to the socket-like transcriptomic profile of the cells, it seems unlikely that a cell type with approximately 2 cells in the first tarsal segment would generate a cluster of equivalent size to the CG43394+ population. For comparison, this cluster included 69 cells, compared to the 84, 74, and 76 in each of the putative sex comb sockets and shafts and chemosensory sheaths, respectively (approximately 11 of each are found in a single ta1). (PDF) [file pbio.3002148.s017.pdf]

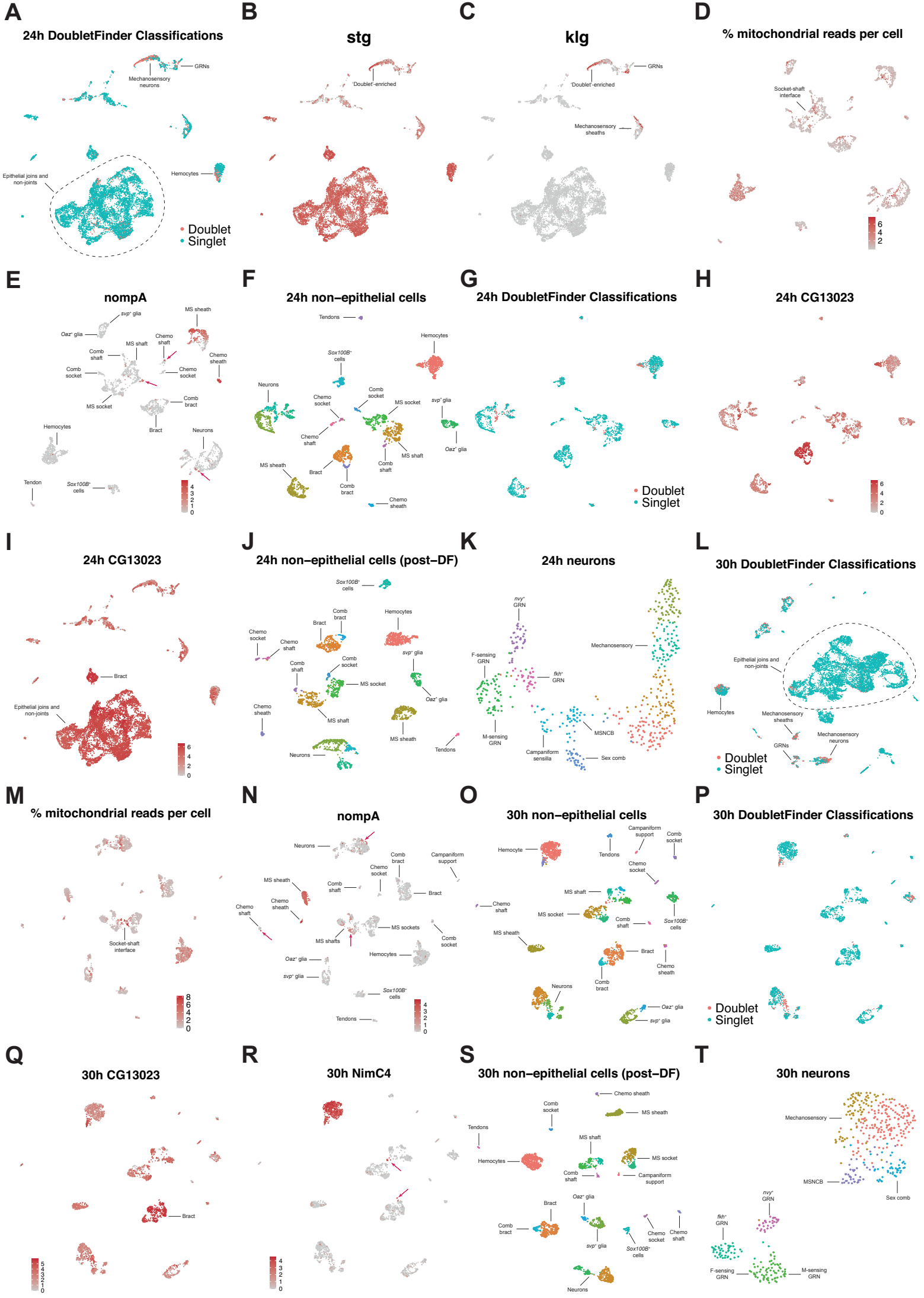

Supplement: S18 Fig — (A) A UMAP plot of the 24 h dataset after low-quality cells have been removed (i.e., those that fail to meet the criteria of >450 genes/cell, <5,000 genes/cell, >2,500 transcripts/cell, <10% mitochondrial reads/cell). Putative doublets identified by DoubletFinder are colored red and singlets blue. Note how in these data, Doublets are heavily enriched within a subregion of mechanosensory neurons and hemocytes. Approximately 29% of cells in the mechanosensory neuron cluster were labeled as doublets. (B) The UMAP plot shown in (A) overlaid with the expression of the mitotic marker stg. Note how the same region of the mechanosensory neuron cluster that is enriched for “doublets” is also enriched for stg. (C) The UMAP plot shown in (A) overlaid with the expression of klg, a gene we identified as one of the top markers of the “doublets” when compared to other mechanosensory cells in the cluster. The only other regions of the UMAP where klg expression is detected is in closely associated subsets of mechanosensory sheaths and gustatory receptor neurons (GRNs). By itself, this restricted expression pattern suggests that these cells are not bona fide doublets. Rather, and considered alongside the stg expression, it suggests that these cells are early differentiating neurons. Given that neurons and sheaths are formed from the same cell division, it further suggests that klg is an early marker of both cell types. (D) The percentage of reads per cell that map to mitochondrial genes overlaid on a UMAP of all the 24 h nonepithelial cells. Note the presence of high % cells at the interface between the socket and shaft clusters. (E) The expression of the sheath marker nompA overlaid on a UMAP of all the 24 h nonepithelial cells. Note the presence of nompA+ cells in other sensory support cell clusters (shafts and neurons; see pink arrows). The presence of nompA is likely indicative of doublets arising through incomplete dissociation of these tightly associated cells within sensory br [file pbio.3002148.s018.pdf]
