# Supplementary material for: A single-cell atlas of the sexually dimorphic Drosophila foreleg and its sensory organs during development
Source: PLoS Biol. 2023 Jun 28;21(6):e3002148. doi: 10.1371/journal.pbio.3002148 (PMC10335707; doi:10.1371/journal.pbio.3002148)
Supplement: S12 Fig — (A) A UMAP showing the annotated GRN clusters identified in the integrated pupal neuron data. (B) The UMAP shown in (A) overlaid with the expression of trol, which encodes the extracellular matrix proteoglycan Perlecan. (C) A UMAP showing the annotated GRN clusters identified in the Fly Cell Atlas single-nuclei adult male leg neuron data. (D) The UMAP shown in (C) overlaid with the expression of trol. (E-G) Confocal images of 30 h APF male first tarsal segments from trol-GAL4 > UAS-mCD8::GFP (green) counterstained with anti-Pros (magenta). We imaged at 30 h APF because at 24 h APF, trol-GAL4 expression in the bristles of the first tarsal segment was either undetectable or very weak. However, at 24 h APF, trol-GAL4 expression was clear in the more distal segments, suggesting these bristles may develop ahead of those in the more proximal segments. The imaged region contains 5 chemosensory bristles, which are circled and numbered in (E). anti-Pros marks 5 nuclei (marked with an asterisk) in each bristle: 4 correspond to GRNs and 1 to the sheath. Individual Pros+ nuclei are visible outside of the circled chemosensory bristles shown in (E). These correspond to the sheath cells of mechanosensory (MS) bristles. In (G), note how there is between-bristle variation in the number of trol-GAL4+ cells. Bristle 1: 2 strongly positive, 1 weakly positive, 2 negative; Bristle 2: 2 positive, 3 negative; Bristle 3: 2 positive, 3 negative; Bristle 4: 3 positive, 2 negative; Bristle 5: 3 positive, 2 negative. This variability matches the expression profile shown in the UMAPs (B, D), where F-sensing and fkh+ GRNs show strong trol expression and M-sensing GRNs show variable expression. Data and code for generating the scRNA-seq elements of this figure are available at https://www.osf.io/ba8tf. (PDF) [file pbio.3002148.s012.pdf]

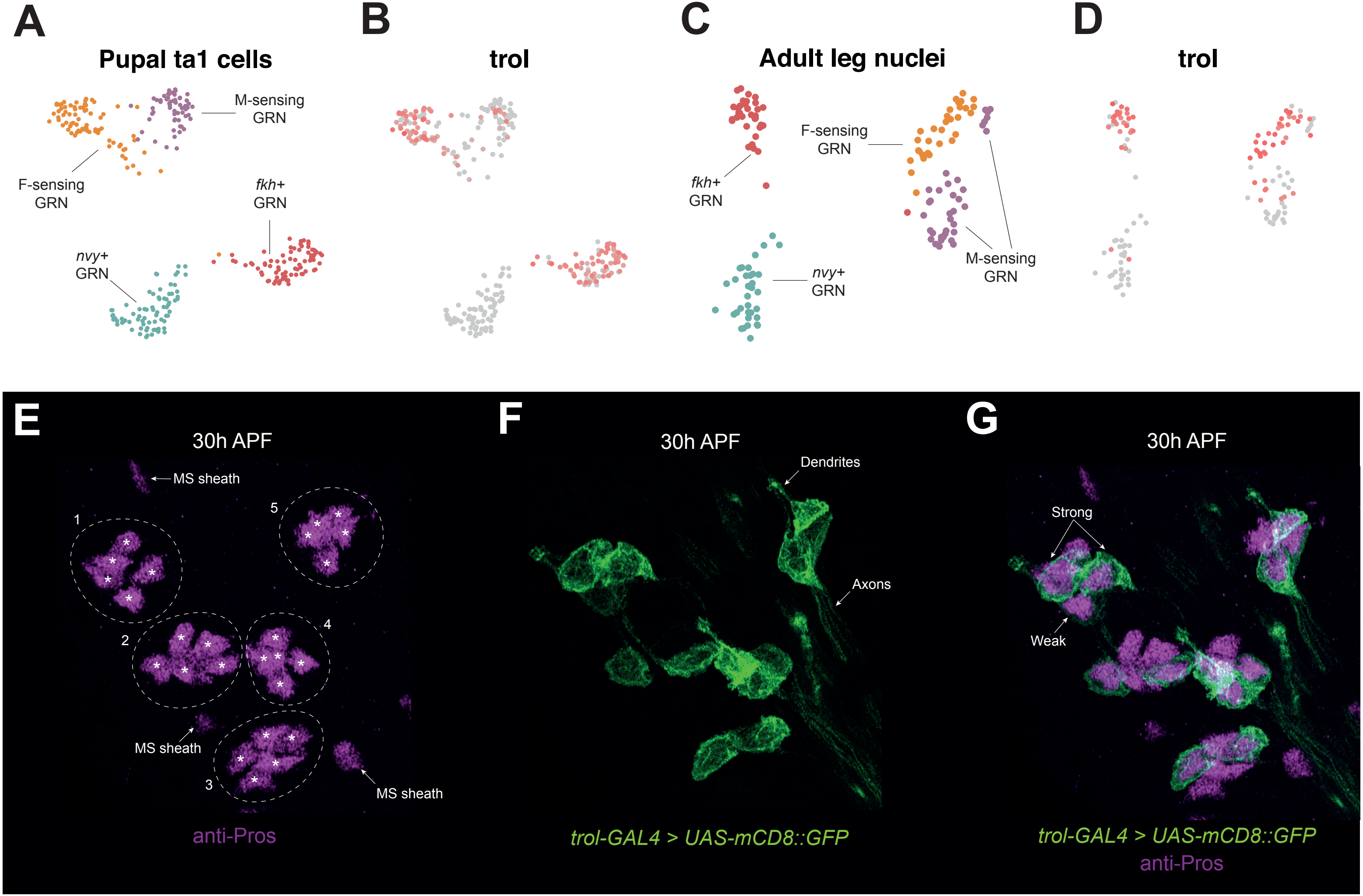
A
Pupal ta1 cells
M-sensing GRN
F-sensing GRN
fkh+ GRN
nvy+ GRN
B
trol
C
Adult leg nuclei
F-sensing GRN
fkh+ GRN
nvy+ GRN
M-sensing GRN
D
trol
E
30h APF
MS sheath
1
2
3
4
5
MS sheath
MS sheath
anti-Pros
F
30h APF
Dendrites
Axons
trol-GAL4 > UAS-mCD8::GFP
G
30h APF
Strong
Weak
trol-GAL4 > UAS-mCD8::GFP
anti-Pros
