# Supplementary material for: A single-cell atlas of the sexually dimorphic Drosophila foreleg and its sensory organs during development
Source: PLoS Biol. 2023 Jun 28;21(6):e3002148. doi: 10.1371/journal.pbio.3002148 (PMC10335707; doi:10.1371/journal.pbio.3002148)
Supplement: S13 Fig — (A) Annotated UMAP of the pupal integrated neuron data. GRN, gustatory receptor neuron; MSNCB, mechanosensory neuron in chemosensory bristle. (B) Annotated UMAP of male neuronal cells subsetted from the Fly Cell Atlas single nuclei RNA-seq leg dataset [38]. (C-N) The UMAPs described in (A) and (B) overlaid with a selection of genes identified among the top markers of campaniform sensilla neurons in the pupal data and which show a loss of specificity when moving from the pupal to adult dataset. (O-T) The UMAPs described in (A) and (B) overlaid with a selection of genes identified among the top markers of MSNCBs and/or sex comb neurons in the pupal data and which show a loss of specificity when moving from the pupal to adult dataset. Data and code for generating this figure are available at https://www.osf.io/ba8tf. (PDF) [file pbio.3002148.s013.pdf]

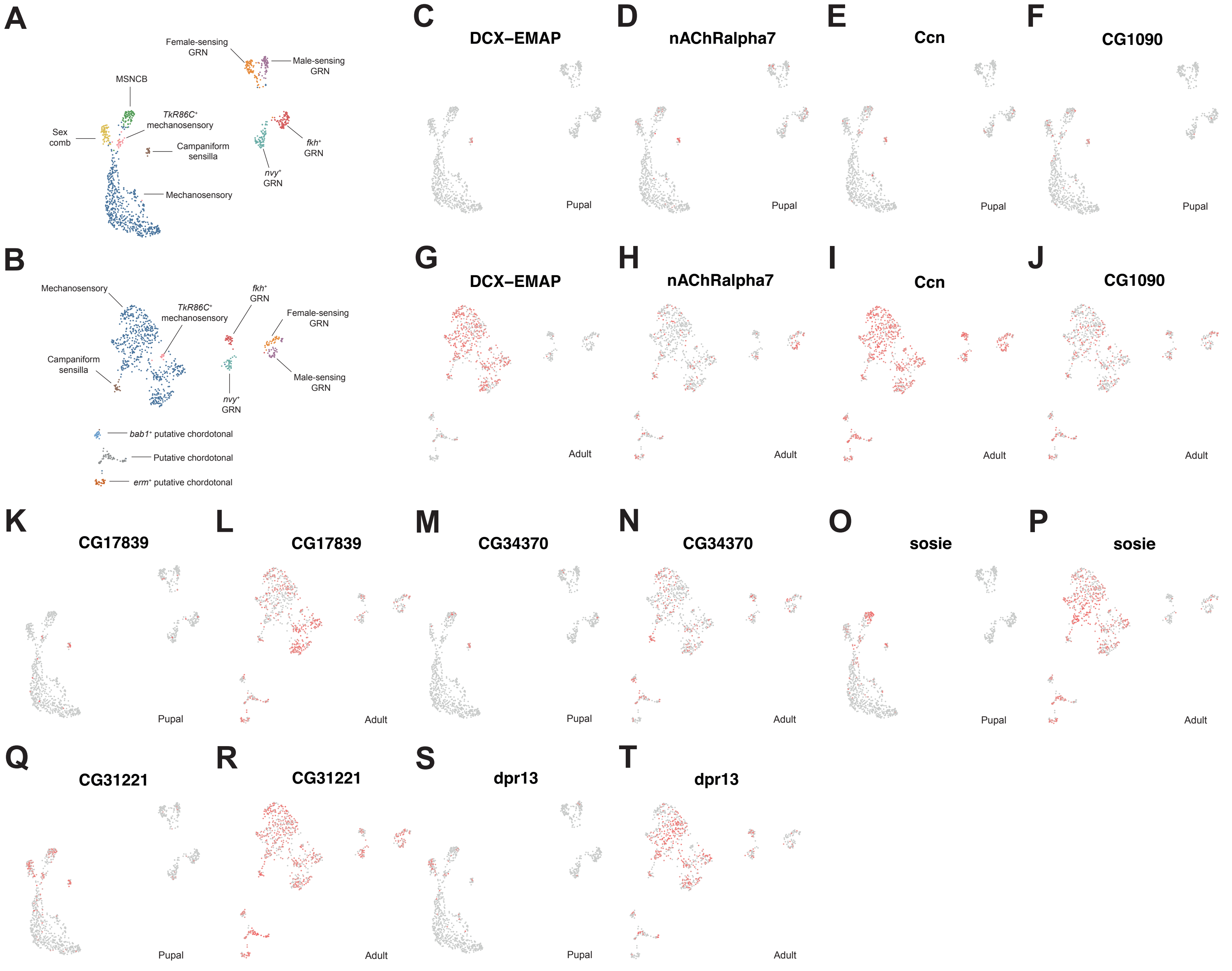

A
Female-sensing GRN
Male-sensing GRN
MSNCB
TkR86C+ mechanosensory
Sex comb
Campaniform sensilla
fkh+ GRN
nvy+ GRN
Mechanosensory
B
Mechanosensory
TkR86C+ mechanosensory
fkh+ GRN
Female-sensing GRN
Campaniform sensilla
Male-sensing GRN
nvy+ GRN
bab1+ putative chordotonal
Putative chordotonal
erm+ putative chordotonal
C DCX−EMAP Pupal
D nAChRalpha7 Pupal
E Ccn Pupal
F CG1090 Pupal
G DCX−EMAP Adult
H nAChRalpha7 Adult
I Ccn Adult
J CG1090 Adult
K CG17839 Pupal
L CG17839 Adult
M CG34370 Pupal
N CG34370 Adult
O sosie Pupal
P sosie Adult
Q CG31221 Pupal
R CG31221 Adult
S dpr13 Pupal
T dpr13 Adult
