# Supplementary material for: A single-cell atlas of the sexually dimorphic Drosophila foreleg and its sensory organs during development
Source: PLoS Biol. 2023 Jun 28;21(6):e3002148. doi: 10.1371/journal.pbio.3002148 (PMC10335707; doi:10.1371/journal.pbio.3002148)
Supplement: S14 Fig — (A) Annotated UMAP of the pupal integrated neuron data. GRN, gustatory receptor neuron; MSNCB, mechanosensory neuron in chemosensory bristle. (B) Annotated UMAP of male neuronal cells subsetted from the Fly Cell Atlas single-nuclei RNA-seq leg dataset [38]. (C-AJ) The UMAPs described in (A) and (B) overlaid with a selection of genes showing enriched expression in the different external sensory organ neuron classes involved in mechanotransduction, namely mechanosensory neurons, MSNCBs, sex comb neurons, and campaniform sensilla. (A-M) Genes identified as top markers of mechanosensory neurons in the adult FCA data, but all show expression in other populations. (N-Z) Genes identified as top markers of campaniform sensilla neurons in the adult FCA dataset. Note how many are also expressed in chordotonal organ populations, but few or no mechanosensory neuron populations. (AA-AB) Across both datasets, Ets65A appears largely restricted to mechanosensory neurons, MSNCBs, sex comb neurons, and campaniform sensilla. (AC-AD) Although relatively widely expressed in the adult data, shakB show marked enrichment in sex comb neurons in the pupal data. (AE-AH) CG42566 and, to a lesser extent, CG33639 appear enriched in MSNCBs. (AI-AL) The 2 effectors of sexual differentiation, fru and dsx, show distinct expression profiles from one another. Data and code for generating this figure are available at https://www.osf.io/ba8tf. (PDF) [file pbio.3002148.s014.pdf]

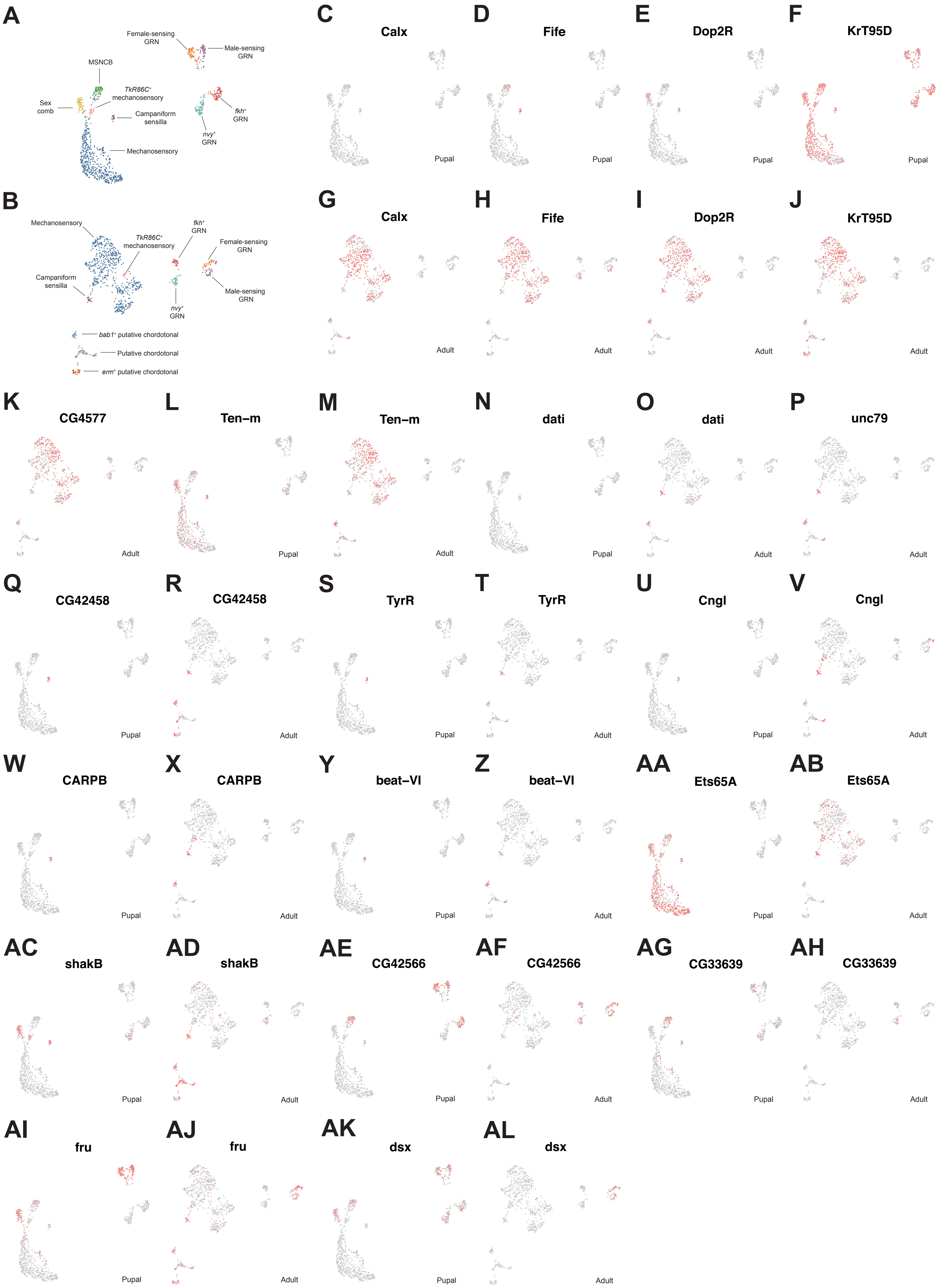

A
Female-sensing GRN
Male-sensing GRN
MSNCB
Sex comb
TkR86C+ mechanosensory
Campaniform sensilla
fkh+ GRN
nvy+ GRN
Mechanosensory
B
Mechanosensory
TkR86C+ mechanosensory
fkh+ GRN
Female-sensing GRN
Campaniform sensilla
nvy+ GRN
Male-sensing GRN
bab1+ putative chordotonal
Putative chordotonal
erm+ putative chordotonal
C Calx Pupal
D Fife Pupal
E Dop2R Pupal
F KrT95D Pupal
G Calx Adult
H Fife Adult
I Dop2R Adult
J KrT95D Adult
K CG4577 Adult
L Ten−m Pupal
M Ten−m Adult
N dati Pupal
O dati Adult
P unc79 Adult
Q CG42458 Pupal
R CG42458 Adult
S TyrR Pupal
T TyrR Adult
U Cngl Pupal
V Cngl Adult
W CARPB Pupal
X CARPB Adult
Y beat−VI Pupal
Z beat−VI Adult
AA Ets65A Pupal
AB Ets65A Adult
AC shakB Pupal
AD shakB Adult
AE CG42566 Pupal
AF CG42566 Adult
AG CG33639 Pupal
AH CG33639 Adult
AI fru Pupal
AJ fru Adult
AK dsx Pupal
AL dsx Adult
