# Supplementary material for: A single-cell atlas of the sexually dimorphic Drosophila foreleg and its sensory organs during development
Source: PLoS Biol. 2023 Jun 28;21(6):e3002148. doi: 10.1371/journal.pbio.3002148 (PMC10335707; doi:10.1371/journal.pbio.3002148)
Supplement: S16 Fig — (A) A subset of the sensory support cell UMAP shown in Fig 8B showing only the mechanosensory and chemosensory sheath populations. Cells are colored in relation to their dataset of origin and therefore, by extension, the time point after puparium formation at which they were collected. (B-J) The UMAP shown in (A) overlaid with the expression of genes in the mechanosensory sheath cluster that we identified as being significantly up-regulated in cells from the 30 h dataset compared to those from the 24 h dataset. Data and code for generating this figure are available at https://www.osf.io/ba8tf. (PDF) [file pbio.3002148.s016.pdf]

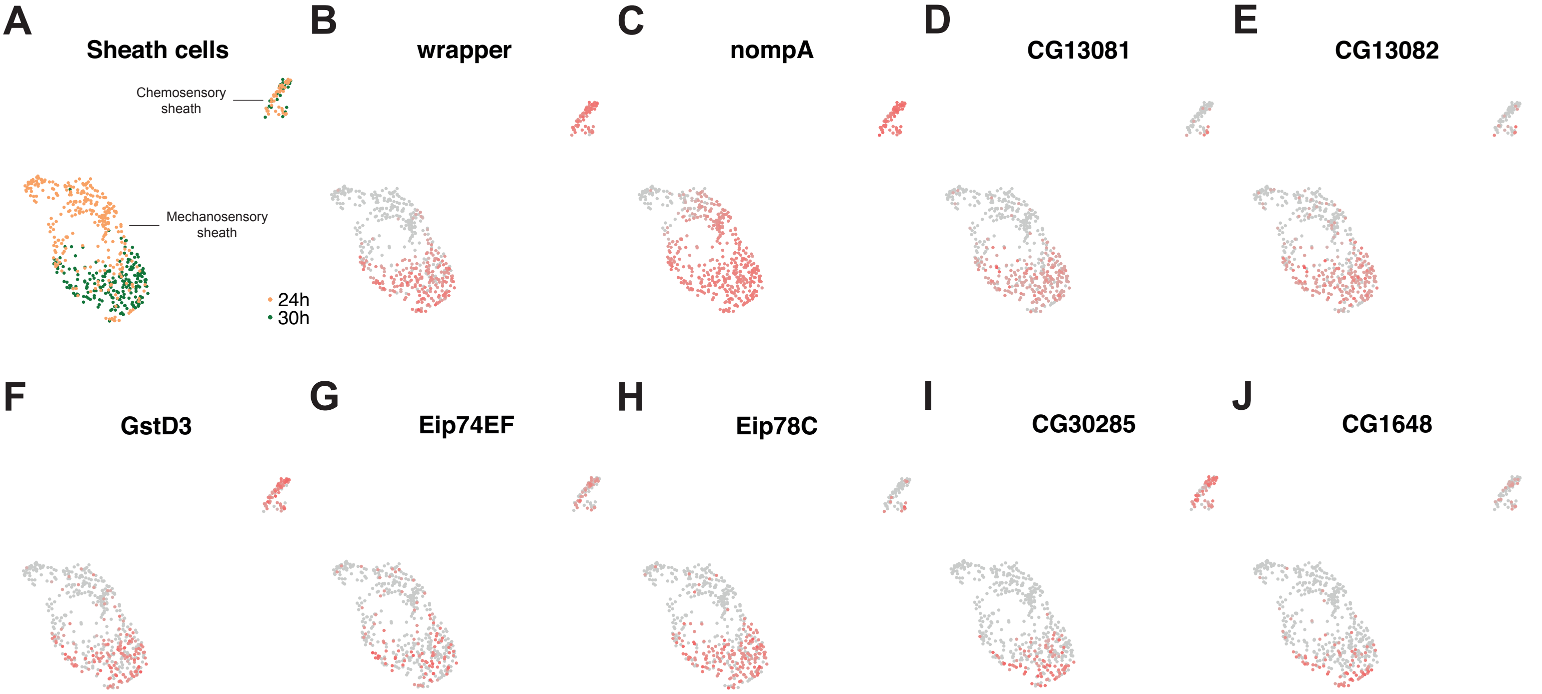
A
Sheath cells
Chemosensory sheath
Mechanosensory sheath
24h
30h
B
wrapper
C
nompA
D
CG13081
E
CG13082
F
GstD3
G
Eip74EF
H
Eip78C
I
CG30285
J
CG1648
