# Supplementary material for: A single-cell atlas of the sexually dimorphic Drosophila foreleg and its sensory organs during development
Source: PLoS Biol. 2023 Jun 28;21(6):e3002148. doi: 10.1371/journal.pbio.3002148 (PMC10335707; doi:10.1371/journal.pbio.3002148)
Supplement: S7 Fig — (A) A dot plot of the top differentially expressed genes from between-glia comparisons (e.g., Oaz+/repo+ glia vs. svp+/repo+ and Sox100B+ glia, etc.). (B) L2 larval brain, ventral nerve cord (VNC), and leg disc from a repo-GAL4 > UAS-mCherry.nls (magenta) male counterstained with anti-Sox100B (green). An inset is shown with a close-up of the disc. Note the staining running along the anterior–posterior axis of the VNC and extending up into the brain. In the VNC, this staining falls along the boundary of the neuropil (see S7D Fig). (C) As in (B) but an L3 larval leg disc. (D) The posterior end of the VNC from a 0 h after puparium formation (APF) male stained with both anti-Sox100B (green) and anti-Futsch (magenta), a neuronal marker. Note how anti-Sox100B staining hugs the outer boundary of the neuropil. Data and code for generating the scRNA-seq elements of this figure are available at https://www.osf.io/ba8tf. (PDF) [file pbio.3002148.s007.pdf]

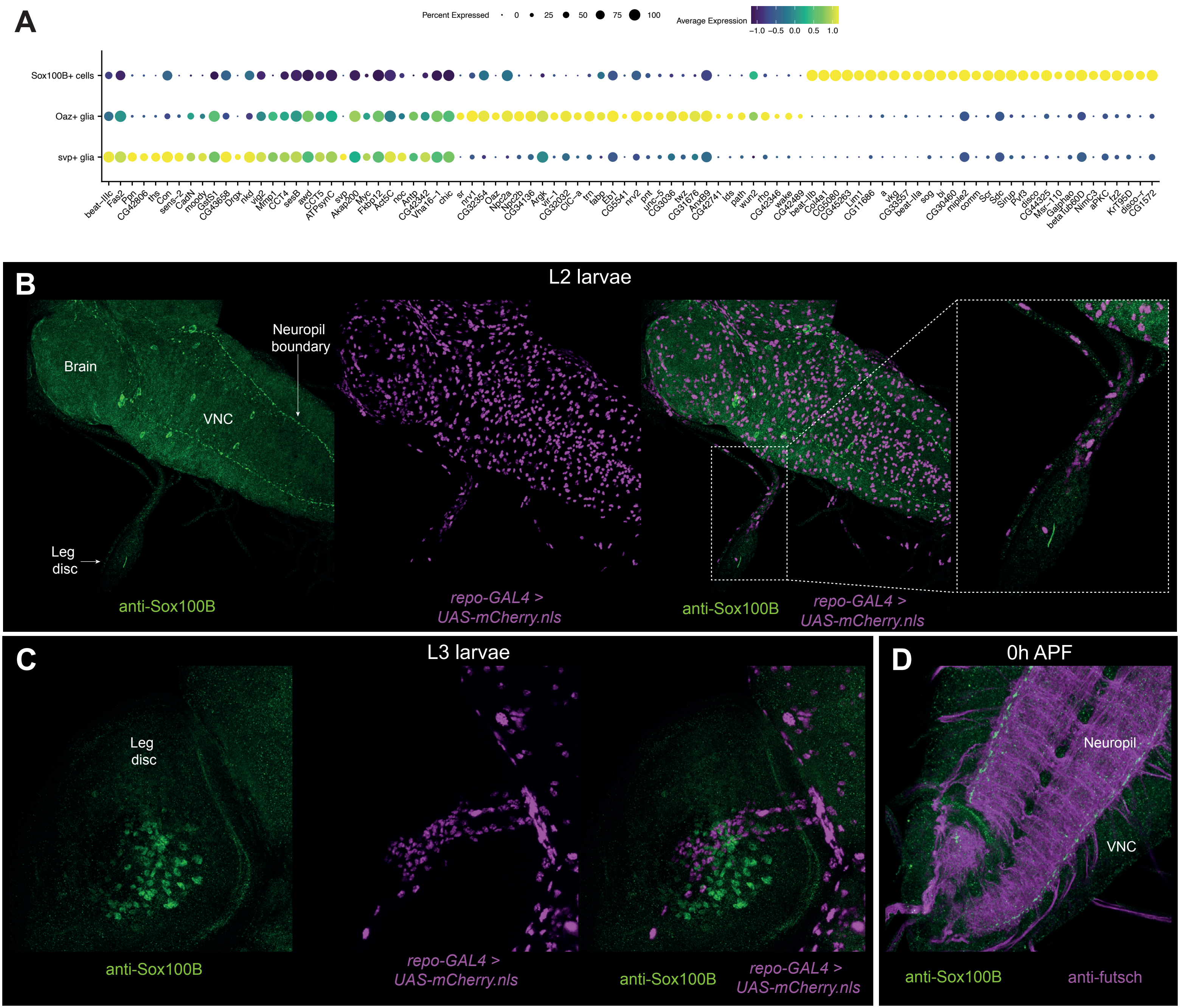

A
Percent Expressed
0
25
50
75
100
Average Expression
−1.0 −0.5 0.0 0.5 1.0
Sox100B+ cells
Oaz+ glia
svp+ glia
beat−IIIc Fas2 Ppn CG42806 ths Con sens−2 CadN moody GstS1 CG43658 Drgx nkd vig2 Mmp1 CCT4 sesB awd CCT5 ATPsynC svp Akap200 Myc Fkbp12 Act5C noc Antp CG42342 Vha16−1 chic sr nrv1 CG32354 Oaz Npc2a Npc2b CG34136 Argk vir−1 CG32032 ClC−a trn fabp Eb1 CG5541 nrv2 pnt unc−5 CG3036 twz CG31676 AnxB9 CG42741 lds path wun2 rho CG42346 wake CG42489 beat−IIb Col4a1 CG5080 CG45263 Lim1 CG11686 a vkg CG33557 beat−IIa sog bi CG30460 miple2 comm Scr Sdc Sirup Pvf3 disco CG44325 Msr−110 Galphao betaTub60D NimC3 aPKC fz2 KrT95D disco−r CG1572
B
L2 larvae
Brain
VNC
Neuropil boundary
Leg disc
anti-Sox100B
repo-GAL4 > UAS-mCherry.nls
anti-Sox100B
repo-GAL4 > UAS-mCherry.nls
C
L3 larvae
Leg disc
anti-Sox100B
repo-GAL4 > UAS-mCherry.nls
anti-Sox100B
repo-GAL4 > UAS-mCherry.nls
D
0h APF
Neuropil
VNC
anti-Sox100B
anti-futsch
