# Supplementary material for: A single-cell atlas of the sexually dimorphic Drosophila foreleg and its sensory organs during development
Source: PLoS Biol. 2023 Jun 28;21(6):e3002148. doi: 10.1371/journal.pbio.3002148 (PMC10335707; doi:10.1371/journal.pbio.3002148)
Supplement: S15 Fig — (A) Annotated UMAP of the pupal integrated neuron data. GRN, gustatory receptor neuron; MSNCB, mechanosensory neuron in chemosensory bristle. (B) Annotated UMAP of male neuronal cells subsetted from the Fly Cell Atlas single-nuclei RNA-seq leg dataset [38]. (C-V) UMAPs of the pupal and adult neurons overlaid with the expression of genes identified as being enriched in the putative chordotonal clusters in the adult data. Note that of these, only CG3339, CG9650, CG43689, and yuri were present in the pupal neuron dataset. (C-K) CG9492, CG10185, CG13133, CG9650, nan, CG14342, Hsc70-2, and CG43149 were found across all putative chordotonal organ cells and largely or entirely absent from the pupal ta1 neuron data. (L) Dhc93AB was present in all chordotonal clusters except for the bab1+ population. (M) NPFR was present in all chordotonal clusters except for the erm+ population. (N-P) The population enriched for the expression of the transcription factor erm showed specific expression of CG14693, Dhc98D, and CG13408. (Q, R) Among chordotonal populations, the transcription factor yuri was largely restricted to the erm+ cluster but also showed expression in GRNs. (S-V) The bab1+ population showed enriched expression of CG3339 and CG43689, although both genes showed spatially restricted expression in a subset of cells in the major chordotonal cluster. Data and code for generating this figure are available at https://www.osf.io/ba8tf. (PDF) [file pbio.3002148.s015.pdf]

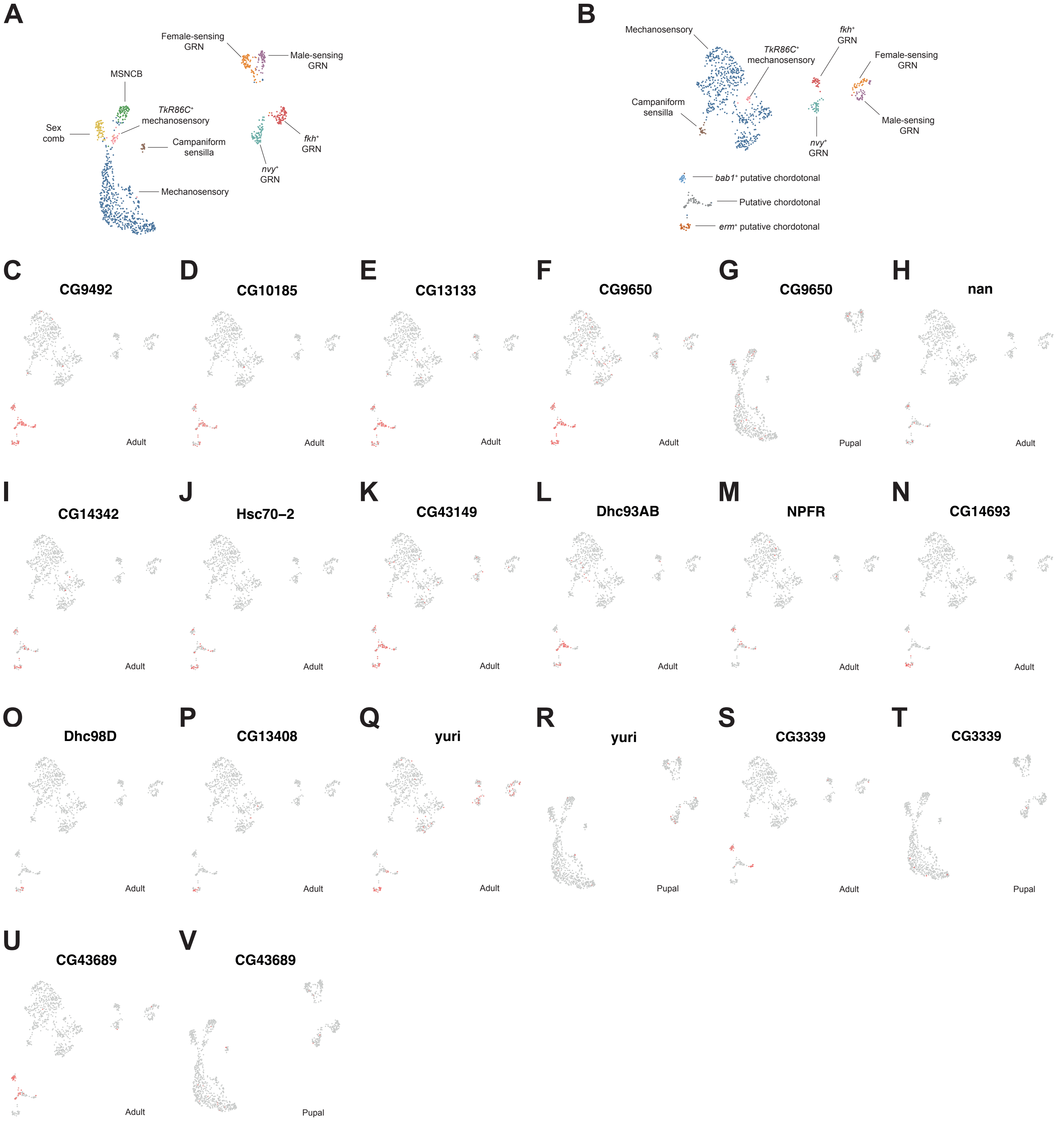

A
Female-sensing GRN
Male-sensing GRN
MSNCB
TkR86C+ mechanosensory
Sex comb
Campaniform sensilla
fkh+ GRN
nvy+ GRN
Mechanosensory
B
Mechanosensory
TkR86C+ mechanosensory
fkh+ GRN
Female-sensing GRN
Campaniform sensilla
Male-sensing GRN
nvy+ GRN
bab1+ putative chordotonal
Putative chordotonal
erm+ putative chordotonal
C
CG9492
Adult
D
CG10185
Adult
E
CG13133
Adult
F
CG9650
Adult
G
CG9650
Pupal
H
nan
Adult
I
CG14342
Adult
J
Hsc70–2
Adult
K
CG43149
Adult
L
Dhc93AB
Adult
M
NPFR
Adult
N
CG14693
Adult
O
Dhc98D
Adult
P
CG13408
Adult
Q
yuri
Adult
R
yuri
Pupal
S
CG3339
Adult
T
CG3339
Pupal
U
CG43689
Adult
V
CG43689
Pupal
