# Supplementary material for: A single-cell atlas of the sexually dimorphic Drosophila foreleg and its sensory organs during development
Source: PLoS Biol. 2023 Jun 28;21(6):e3002148. doi: 10.1371/journal.pbio.3002148 (PMC10335707; doi:10.1371/journal.pbio.3002148)
Supplement: Attachment — Submitted filename: Response_to_reviewersv2.1.docx [file pbio.3002148.s024.docx]

To the editor,

We thank the editors and reviewers for their thoughtful remarks, kind words about our paper’s strengths, and for identifying the areas that needed clarification or improvement. We believe that their suggestions have substantially improved the manuscript. We provide a point-by-point response to each of the reviewers’ comments below. In addition to these reviewers’ comments, we have made several further changes to address points raised in the decision letter. First, we have reformatted our in-text references to supplementary figures to bring them in line with standard PLOS usage. Second, in line with the PLOS protocols deposition policy, we have now published a full, detailed protocol for generating single cell suspensions of pupal tarsus tissue to protocols.io. This can be viewed via the following link [dx.doi.org/10.17504/protocols.io.x54v9dzbmg3e/v1](x-webdoc://555282E4-B003-4965-A772-18750C7C4649/dx.doi.org/10.17504/protocols.io.x54v9dzbmg3e/v1). We have also made a small number of edits to the main text of the manuscript to improve readability. All changes can be viewed as tracked changes in the revised manuscript text.

Thank you for considering our article for publication in PLOS Biology.

Best wishes,

Ben Hopkins, Olga Barmina, and Artyom Kopp.

------------------------------------------------------------------------------------------------------------------------------------------------------

Dear Dr Hopkins,

Thank you for your patience while your manuscript entitled "Charting the development of Drosophila leg sensory organs at single-cell resolution" went through peer-review at PLOS Biology. Your manuscript has now been evaluated by the PLOS Biology editors, an Academic Editor with relevant expertise, and by three independent reviewers.

In light of the reviews (attached below), we are pleased to offer you the opportunity to address the comments from the reviewers in a revision that we anticipate should not take you very long - we will leave up to you to perform the experiments suggested by Reviewer 3. We will then assess your revised manuscript and your response to the reviewers' comments with our Academic Editor aiming to avoid further rounds of peer-review.

In addition, we would like to consider your manuscript as a Methods and Resources manuscript, as we think it will fit better this format, thus please select this article type when you submit your revision.

We expect to receive your revised manuscript within 1 month. Please email us ([plosbiology@plos.org](mailto:plosbiology@plos.org)) if you have any questions or concerns, or would like to request an extension. 

At this stage, your manuscript remains formally under active consideration at our journal; please notify us by email if you do not intend to submit a revision so that we withdraw the manuscript.

------------------------------------------------------------------

Reviewers' comments

Rev. 1:
This manuscript presents a thorough description of cell types that occupy the first tarsal segment of the Drosophila foreleg during pupal development (stages 24h and 30h after pupal formation), in an effort to understand what differentiates the different cell types and how these differences arise during development. Hopkins et al. performed single-cell sequencing of this tissue at the mentioned time points and described the different cells types.

- They first divided the cells into epithelial and non-epithelial cells, as the epithelial cells were very abundant; they discerned the joint cells from the rest of the epithelial cells and identified their transcriptomic signature. They then looked at the rest of the epithelial cells; very interestingly, they saw in the UMAP representation that epithelial cells were organized according to their anatomical position along the anteroposterior, dorsoventral, and proximodistal axes. They found differentially expressed genes that were already known to characterize these axes, as well as new marker genes.

- They then looked into the non-epithelial non-sensory cells, i.e. the hemocytes, glia, and bracts. There, they had a number of interesting observations: first, they noticed that bract cells, despite their epithelial origin, are very different from epithelial cells, concluding that, once committed to a bract identity, the epithelial cells change their transcriptome drastically. Second, and more interestingly, they identified a novel cell type not expressing repo, but resembling glia in many regards (e.g position), that seems to be related with the neural lamella, as it expresses a lamella marker gene (vkg).

- Then, the authors looked into the sensory neurons where they find a very neat combinatorial transcription factor code that discerns all sensory neurons. A lot of work is very well summarized in Figure 9. They use their clustering and annotation to go into detail in the different cell types to study the genes and receptors that differentiate them. One interesting observation that the authors made is that many genes that they find being cluster-specific in pupal cell types are not specific in adult neurons (for probing adult neuron expression, they use the recently published Fly Cell Atlas data). They attribute this to heterochrony, arguing that different neuronal types are at different stages of their differentiation path leading to stage-specific differences that are alleviated when the neurons are fully differentiated.

- Finally, they focused into the support cells (shaft, socket, and sheath) of the different organs, where they found similarities and differences in the expression patterns of homologous support cells of different organs.

In general, the manuscript is very thorough and detailed. It has observations that uncover very interesting biology (such as the Sox100B+ cell type that seems to be associated with the neural lamella) and open new avenues of research. The Introduction is very well written (for non-leg experts, such as myself) and the Discussion is very interesting and stimulating. The Results are very detailed (maybe too detailed for non-aficionados), there is a lot of work and information in a fairly long paper. Importantly, the results are carefully and justly interpreted. The Methods are also very detailed. I think that this paper will be a great addition to the literature and will be very helpful to many future studies. I think it is one of the best written atlas papers I have read.

We thank the reviewer for their kind words.

Having said the above, I believe that the title is exaggerating the findings (or even the experimental setup) of the paper. Profiling these two stages is definitely not sufficient to "chart the development of Drosophila leg sensory organs". I would re-phrase the title to make it more accurate. Similarly, in the abstract, the authors claim to address the question of how this diversity is generated during development, but I don't think that they really address it.

We understand the author’s concerns here. In relation to the title, we have how changed it to: ‘A single-cell atlas of the sexually dimorphic *Drosophila* foreleg and its sensory organs during development’.

We have also now rephrased the wording in the abstract to minimize any claims about identifying specifically developmental drivers, instead placing the emphasis on resolving genetic differences between cell types. The sentence now reads: ‘To understand the genetic basis of this diversity of cell types, both within and between sensory organs, we performed single-cell RNA sequencing on…’

Also, when the authors describe the Sox100B+ cells, they claim that these cells appear to originate with the leg disc itself (line 319). Having seen only static images, it is very hard to conclude this. If the authors want to keep this point, they should try to do live imaging or, at least, a timelapse.

We thank the reviewer for this suggestion. We have now repeated these stainings across both early- and late-stage L2 and L3 larvae. Representative images are now included in S7 Fig and new text added to our section ‘*A novel cell type associated with the neural lamella’.* These stainings provided further support for our original claim that Sox100B^+^ cells do not migrate into the disc from the central nervous system, as do many glia cells, but rather originate within the disc itself. Across both L2 and L3 larvae, we observe clear migration of *repo-GAL4^+^* cells into the leg disc. In contrast, Sox100B^+^ cells are only visible within the disc in L3 larvae and at no point do we see evidence of them migrating into the disc.

These same experiments, along with a new double staining of anti-futsch (a neuronal marker) and anti-Sox100B, also more clearly revealed the distribution of Sox100B cells in the VNC. A glia-like association with the neuropil of the VNC is clear in images S7B and S7D, mirroring the associations we detect between these cells and the axon trunks of the leg.

I also have some minor comments, which, I believe, could improve the manuscript, but I acknowledge that there is already enormous amount of work in there, so they are not necessary to be addressed.

- Line 29: "organ" should be "organs"

Thank you. This has been corrected.

- Intuitively, GRNs remind me of Gene Regulatory Networks and I was initially confused, although the authors define it immediately. If it is not an already established abbreviation, it might help to change it a bit.

As primarily developmental geneticists, we understand the confusion. However, ‘GRN’ is an established and widely used abbreviation in the Drosophila neuro literature. We’ve opted to stick with ‘GRN’ to avoid confusing that audience. To make clear our meaning, we now define GRN several times throughout the manuscript: on first use in the introduction, results, discussion, and methods; in the first section title that features ‘gustatory receptor neuron’; and in each figure legend.

- In line 98, the authors argue reasonably that "techniques and approaches that help overcome these barriers are of significant value". Reading the manuscript and the Methods, it was unclear to me what the authors did differently that enhanced the recovery of the cells that are associated with the cuticle.

We have slightly changed the phrasing quoted here by the reviewer and added an extra sentence at this stage that introduces our approach. This section now reads: ‘Consequently, approaches that help characterize the transcriptomes of cuticle-associated cells are of significant value. In principle, the developmental window between pupa-adult apolysis – the separation of the pupal cuticle from the epidermis – and the formation of the adult cuticle (~12-48h after puparium formation, APF; Reed, Murphy, & Fristrom, 1975) provides a rare opportunity during which such cells might be accessible.’

- In Figure 2 - Figure supplement 1B,D, it would be good if the y-axes were the same between the two samples for easier comparison.

We thank the reviewer for their suggestion, which we have now implemented.

- In the section where the epithelial cells are annotated based on their anatomical position, the dorsoventral axis overlaps with the proximodistal (both are on the x-axis). It might worth trying a 3D UMAP, in case this resolves the three axes.

This is a clever idea from the reviewer. However, having now tried this approach, we find that it doesn’t make for a significant improvement in the separation of the anatomical axes along xyz axes; if anything, the static 3D visualizations are less clear than the 2D originals.

- Line 240: "thus, these cells likely correspond to sex comb bracts." An antibody staining could resolve this.

Unfortunately, we were unable to identify any reagents to perform this experiment, as there are no unique sex comb markers. *rn* and *dsx* are both expressed in the wider body of epithelial cells surrounding the sex comb, so using these to distinguish them from the bracts would be a challenge. However, we believe that the evidence on which we’re inferring a sex comb bract identity for these cells is strong: they express the documented bract markers *aos* and *pnt* (del Álamo, Terriente, & Díaz-Benjumea, 2002; Peng, Han, & Axelrod, 2012; Mou *et al.*, 2012), are closely associated with the mechanosensory bract cluster, and express two genes that previous work (Tanaka, Barmina, & Kopp, 2009; Natori *et al.*, 2012) has shown are restricted within the first tarsal segment to the sex comb bearing region (*rn* and *dsx*).

- Line 246-248: This is unclear to me. Does this mean that the support cells are affecting bract cell identity? How? I feel like I am missing something.

We thank the author for pointing out the lack of clarity in this sentence. We have now refocused it on the partially overlapping morphological characteristics of bracts and shafts. The amended sentence now reads: ‘It was common to find expression of some of these genes (*e.g. CG33110, Nep2, CG32365, neur*; S5F-S5P Fig) in bristle shaft and socket cells, which, considering the short bristle-hair like protrusion that bracts develop, may reflect their partially overlapping morphological characteristics.’

- Line 272-273: When is sr usually expressed? An antibody staining or FISH could resolve whether this is indeed due to the developmental stage.

We are unaware of any work on *sr* expression specifically in the leg. And indeed, we were probably wrong to suggest that the surprising absence of *sr* from this tendon cell cluster reflects the ‘relatively early developmental window during which we sequenced these cells’ because the expectation from previous work on flight muscle tendon development and embryonic tendon development is probably that *sr* should be expressed in tendon cells throughout development. This is because although it’s the earliest known tendon marker it is also necessary for terminal differentiation of the tendons after muscle attachment (Frommer *et al.*, 1996; Volohonsky *et al.*, 2007; Wayburn & Volk, 2009). Therefore, it may be that *sr* activity in the tibia/tarsus joint tendons differs from that observed in the more commonly studied tendons, such as those that connect to the wing.

In light of this, we have modified the text in this region, which now reads: ‘Their absence may reflect the developmental timepoints that we sequenced or differences between this tibia/tarsus tendon population and more commonly studied populations in the embryo (Volohonsky *et al.*, 2007) and wings (Fernandes, Celniker, & VijayRaghavan, 1996)’.

While the reviewer is right to suggest that an antibody staining or FISH might help to resolve at what point *sr* begins – and ceases – expression in the tibia/tarsus tendons, the primary focus of this work is on the sensory system, rather than tendons and muscles. Consequently, and in light of the reviewer’s comment that these additional experiments are ‘not necessary to be addressed’, we prioritized other experiments.

- Line 291-293: Could this be a difference between RNA and protein? Maybe FISH could answer the question.

Here, the reviewer is referencing our finding that a number of documented markers of specific glia subpopulations are surprisingly widely expressed among glia subtypes and asks a good question of whether this reflects a discrepancy between RNA and protein levels. This is certainly possible, but our results raise this question on a large scale, since we observe the discrepancy not for one gene but for many of them, suggesting that post-transcriptional regulation could be an important feature of glia development. But the scope of experiments needed to address this hypothesis, which would require time-series FISH coupled with antibody staining for multiple glia markers, is beyond the scope of this already large study. We discuss this potential disconnect between protein and RNA, along with the elevated importance it may play in glia, in a dedicated section in the original discussion.

- Line 332: I am really not a big fan of "data not shown". Could the authors remove this phrase or provide the data or link to a paper?

We are not ready to publish the 16h data here – introducing and describing a third dataset would add length and complexity to an already long and complex paper. That said, we are confident in our detection of separate Sox100B^+^ cells and myoblasts, the latter identifiable based on the joint expression of *Mef2* and *twi* (Cripps *et al.*, 1998). However, we understand the reviewer’s frustration with this sort of language, so have removed it. The anti-Mef2 stainings along with the transcriptomic data we present by themselves provide convincing evidence that Sox100B^+^ cells are not myoblasts. The amended section now reads: ‘To exclude the possibility that these cells correspond to myoblasts, we compared the distribution of Sox100B^+^ cells to those stained by an antibody raised against the myoblast marker Mef2 (S8C Fig)(Cripps *et al.*, 1998). Unlike Sox100B^+^ cells, myoblasts were restricted to the tibia and absent from tarsal segments, arguing against a myoblast identity for Sox100B^+^ cells.’

- Regarding the Sox100B+ cells, the authors argue that these are glia-like cells, although they don't really define what glia is (which is a common problem with glia, as different people define it differently). I was wondering whether the authors looked at other glial markers beyond repo and wrapper.

Generally, we’ve defined glia in relation to documented subtypes within the leg (perineural, subperineural, and wrapping; Sasse & Klämbt, 2016). The exception to this is the ‘Sox100B+ cells’ we identify. In the original draft, we referred to these variously as ‘Sox100B+ cells’ and ‘Sox100B+ glia’. We’ve now changed this to consistently refer to them as ‘Sox100B+ cells’. In the text itself, we refrained from identifying these cells as a glia population, but did describe their ‘glia-like’ behaviour: they express the glia marker *wrapper* and *beaten path* genes, the latter of which play important roles in neuron-non-neuron communication (Li *et al.*, 2017); form close physical associations with neurons; and are patterned along the proximal-distal axis of the leg in a similar way to *repo*+ glia.

As part of our efforts to identify glia subtypes within the leg, we ran through an extensive panel of glia markers. UMAPs of a number of these are presented in Figure 5 and Figure S8, along with dotplots that compare the expression of *bona fide* glia genes (*i.e.,* those identified as top markers of our *repo+* populations) between *repo*+ glia and our *Sox100B+* cells (Figure S7A). Some of these showed appreciable expression in *Sox100B+* cells (*e.g. wrapper,* Figure 5AB; *apt,* Figure 5L). However, given the surprisingly non-specific expression patterns of many glia markers in the first place, the expression of these glia markers didn’t help us to link Sox100B+ cells to a known glia population.

- The section "repo+ glia and Sox100B+ cells express distinct cell-cell communication gene repertoires" is very handwavy and fits more into the Discussion. Similarly, I feel that the section "Heterochrony in the gene expression profiles of sensory organ neurons" is very preliminary and could also go to the Discussion. Otherwise, the authors would have to justify their claims by performing antibody stainings at different stages to prove that there is indeed heterochrony.

We appreciate the reviewer’s concerns here. With respect to the heterochrony section, we have now changed the title to ‘Potential heterochrony in the gene expression profiles of sensory organ neurons’ and trimmed away the more speculative concluding sentences of this section. Nevertheless, these heterochrony-like gene expression changes still present a clear signal in our data, so we feel that it is important to mention them. We’ve also added some text that notes how this pattern of heterochrony is consistent with what we know of the developmental timing differences between sensory organ classes: ‘This hypothesis is consistent with documented between-organ variation in developmental timing: chemosensory bristles are known to be specified earlier than all but the largest mechanosensory bristles (Rodriguez *et al.*, 1990; Held, 1990).’

With respect to the cell-cell communication section, we feel that it works well as is. It’s a relatively short section and describes one of the sharpest differences between the glia and Sox100B+ populations. Indeed, *beat-IIb* was among the top global (*i.e.,* compared to all other cells in the datasets) markers of Sox100B+ cells. The strong association of *beaten path* genes with the developing nervous system feels like an important piece of information to consider alongside the neuronal associations we report for Sox100B+ cells in the previous section.

- In Figures 6N-P, it was unclear to me how the authors identified the sheath cells.

The reviewer is asking how we identified sheath cells in the images of chemosensory bristles presented in Figure 6N-P. We identified these cells based on the fact that they were (a) positive for Pros, a documented canonical marker of sheath cells (Simon *et al.*, 2009), and (b) negative for *elav-GAL4,* a widely used neuronal marker. We have amended the introductory sentences of this paragraph to clarify this, which now reads: ‘Unlike in mechanosensory bristles, we detected multiple Pros^+^ cells per chemosensory bristle at 24h APF (Figure 6N-P). One of these was *elav-GAL4^-^*, consistent both with a sheath identity and, when contrasted against the heterogenous *elav-GAL4* expression we observed among mechanosensory sheaths, the developmental timing differences that are known to exist between mechanosensory and chemosensory bristles: chemosensory bristles are specified earlier than all but the largest mechanosensory bristles (Rodriguez *et al.*, 1990; Held, 1990)’.

- In line 429-430, When is Poxn usually expressed? An antibody staining or FISH could resolve why it was not detected.

We know from antibody staining work previously conducted in our lab that Poxn is present in early differentiating chemosensory bristle cells at 5h after puparium formation (APF) (Luecke, Rice, & Kopp, 2022). It’s expression thereafter is unknown. While we do not find *Poxn* in the 24h and 30h datasets we analyse in this manuscript, we have found that a small population of apparently early differentiating chemosensory neurons express *Poxn* in a 16h APF dataset that we have collected. We are therefore likely too late to detect it in our 24h and 30h datasets. We have added text to the manuscript that discusses that we may be too late for *poxn* expression:’ Surprisingly, we failed to detect *Poxn,* which is known to be both necessary and sufficient for a chemosensory rather than mechanosensory fate, in GRNs in either dataset, which suggests that *Poxn* transcription may have ceased by 24h APF (Bopp *et al.*, 1989; Nottebohm, Dambly-Chaudière, & Ghysen, 1992; Nottebohm *et al.*, 1994; Dambly-Chaudière *et al.*, 1992; Awasaki & Kimura, 1997)’.

- In Figure 7AB, some kind of color-coding of the similarly expressed genes could facilitate the reading of the table.

This is an excellent idea. Genes that are shared between different neuron classes are now colour coded. We have updated the figure legend to explain this, adding ‘Gene names are coloured pink, green, or blue when shared across multiple GRN subtypes.’

- Lines 558-560: "But in the adult dataset these differences were reduced or lost, suggesting that the variation we observed between GRNs in the pupal data may reflect heterochronic differences (Figure 7-figure supplement 2AD-AF)." While this is definitely a valid hypothesis, an alternative is that the cells are indeed different during development and they then converge to a similar identity. This has been shown to be the case in other neuronal systems, such as the visual and olfactory neurons.

This is an interesting idea. We have now incorporated it into the text. This passage now reads: ‘But in the adult dataset these specific differences were reduced or lost, suggesting that the variation we observed between GRNs in the pupal data may reflect heterochronic differences or that these particular between-GRN developmental differences are lost in adulthood’

- Figure 10 seems more appropriate to be in the Supplement.

We agree with the reviewer and have now moved this to the supplementary material.


Rev. 2:
Summary:
In their manuscript "Charting the development of Drosophila leg sensory organs at single-cell resolution" Hopkins et. al improve our understanding of the cell types required to generate the sensory organs in Drosophila tarsi through development. By focusing on the first tarsal segment of the Drosophila pupal foreleg, the authors elucidate the combinatorial transcription factor codes of mechanosensory, sex comb and campaniform sensilla neurons as well as distinguishing between four different gustatory receptor neuron classes. Their work also reveals the receptor and membrane channels found in specific neurons and the transcriptomic differences in the non-neuronal cells in the region, such as tendons, hemocytes, bract cells and glial cells. Additionally they identify a novel cell type that contributes to neural lamella construction. This impressive annotated dataset is also available as a resource for the Drosophila community.

Strengths:
-The authors make excellent use of their developmental timepoint from an academic and experimental perspective. Assaying the first tarsal segment at 24h and 30h APF provides insight into sensory organ specification, while also bypassing the challenge of isolating nuclei protected by the cuticle. By using an enzymatic digestion, the authors are able to improve read depth and reduce gene dropout, benefitting the detection of are cell populations.

-The authors provide a step-by-step understanding of the rationale and process behind their data analysis as they breakdown their datasets by cell type across multiple UMAPS to identify their transcriptional signatures.

-Their work is validated with the use of genetic reagents to confirm the location of the genes and cells being described.

-The comparison to an adult dataset adds to the richness of the resource in tracking sensory organ development in the tarsus.

We thank the reviewer for their kind words.

Questions:

-Based on lines 998-1000, the authors appear to have only included one biological replicate for each of the two timepoints. Is this accurate, and if so, could the authors comment on the rationale behind not including other replicates and the impact they anticipate this may have had on the data? It seems that multiple experimental replicates should be included to provide the basis of a more robust analysis.

Our differential expression analyses were designed to look for genes that were conserved markers of a given cluster (or set of clusters) across two different timepoints separated by 6 hours. This represents a more conservative strategy than identifying genes that were differentially expressed in a cluster across either of the two datasets, which would likely be more prone to spurious results.

As shown in the paper, the two timepoints show very similar clustering patterns and marker gene expression patterns. The consistency in gene expression across cells between the two timepoints that we document supports the accuracy of our cell type identification. Moreover, we used a variety of staining approaches to provide an orthogonal validation for our cell type identifications. It’s possible that with additional datasets – and therefore additional cells – we may have had the resolution to detect rarer cell types, the numbers of which may be too low in our dataset to cluster separately. That said, there are only 3 campaniform sensilla neurons in the first tarsal segment and we were able with just two samples to identify these as a distinct cluster. It’s hard to imagine there being many (any?) cells less abundant than this in the dissected region.

Another point to make is that we did make use of an additional adult dataset to cross-reference our neuronal data against. This served both as a validation of the transcription factor code that we uncovered, as a means of identifying additional GRN-specific genes that may not have been expressed at the early timepoints we sequenced, and as a way of cross-validating our GRN-specific designation of some neuronal genes that were expressed across developmental and adult timepoints.

We did not believe that our two timepoints, each with a single replicate, approach was appropriate for testing for quantitative differences in expression between timepoints in a given cluster. For drawing strong quantitative conclusions about between-timepoint differences, we would want to use a replicated design. We therefore focused our analyses on the more qualitative differences between cell types.

-As mentioned in lines 831-839, one possibility for why the authors were only able to identify 4 GRN populations might be because other distinct GRN classes might exist in other tarsal segments, such as in the taste bristles at the tip of the leg. With this in mind, could the authors clarify why they chose to focus on the first tarsal segment to the possible detriment of the other important, but rare, cell types in the rest of the tarsus?

We thank the reviewer for this excellent question. It is true that by focusing in on the first tarsal segment we are missing out on taste bristles in other parts of the leg. This is why we were keen to incorporate the Fly Cell Atlas leg data, which is generated from dissections of all three pairs of legs, and all segments of each leg. Our recovery of the same four GRN populations in a dataset from a more expansive region of tissue lent weight to our model of a minimal class of core GRN identities. Our decision to sequence only the first tarsal segment was based on our desire to maximize our recovery of sexually dimorphic sensory organs. The first tarsal segment contains both the sex comb and a number of sex-specific chemosensory bristles that appear to be specialized for the detection of pheromones. Increasing the dissected region would reduce our total recovery of these cells as they make up an increasingly small proportion of the total number of isolated cells. Thus, we had to trade off our ability to recover additional taste bristles against maximizing our recovery of sexually dimorphic organs. We describe our decision to focus on this region in the introduction: ‘Using a fine-scale dissection technique, we specifically target the first tarsal segment (the ‘basitarsus’) to maximize the detection of rare sensory organ types, including the campaniform sensilla, chemosensory taste bristles, and sex comb teeth.’

Rev. 3:
This manuscript characterized all major cell types in the male fly foreleg using single-cell RNAseq data from three time points (two from development, generated by this study, and one from adult, published by the FCA project).

This manuscript is probably the most comprehensive paper, I have ever read, for characterizing detailed cell types within a specific tissue. The Authors carefully checked known makers for validation, and also identified new markers, which representing new exciting findings for next studies. For example, they thoroughly compared development and adult data and concluded that there are four types of gustatory receptor neurons. They also identified a novel glia-like cell population which may play important function for the lamella development. All findings are clearly written, and references are properly cited.

I don't have major concerns for this manuscript to publish but have a few suggestions and comments to further improve it.

1. There's no overview of all cell types from the scRNA-seq data, and it's very difficult to follow the analytic logics. I will suggest the authors to re-organize the Fig. 2 into the following orders. First, integrate 24h and 30h datasets and illustrate batch consistency. Second, using one tsne/umap plot to show all broad cell types identified in this study. Marker genes defining each broad cell type should be included in the supplement. Third, show the proportion of the broad cell types from in each dataset, including epithelial joints, epithelial non-joint, neuron, non-sensory, and sensory support cells. Details of different subclustering can be addressed in next figures.

We thank the reviewer for their suggestion. Our goal with this figure is to (a) justify our decision to use an integrated dataset of the two timepoints, and (b) to introduce the various subsetted datasets around which each subsequent figure is framed. We experimented with several designs for this layout, including one similar to what the reviewer is describing, and the one we used in the manuscript was judged by several readers to be the clearest. By detailing the subclustering process up front, we free up both space and words in many of the subsequent figures. In our early versions of this figure, we also found that providing an annotated UMAP of all cells wasn’t particularly helpful as a lot of our smaller subpopulations were hard to discern at this scale.

2. Fig. 2A-J, since 24h and 30h data can be integrated well, cell-types markers can be shown in integrated data.

Our intention with these separate timepoint figures was to justify our decision to integrate the datasets by showing that populations marked by the same classical marker genes were present in both datasets. All of these markers are shown in integrated, subsetted datasets across the subsequent figures.

3. Fig 3A and 3C are redundant. Fig 3B and Fig3D are also redundant.

We thank the reviewer for their comments. However, we respectfully disagree on this point. There are two senses in which the reviewer could mean redundant: either overall or with respect to each other (*i.e.,* only one of Fig. 3A or 3C is required). First, we will argue that they are not redundant overall.

Figures 3B and 3D show the distribution of two of the top markers (*nub* and *TfAP-*2) of the two regions of the epithelial cell UMAP that cluster separately (which we later show correspond to joints). Figure 3B shows these genes overlaid on the full epithelial cell UMAP to emphasize that they are specifically enriched within joint clusters. Figure 3D then shows them overlaid on the joint UMAP. Key here is the sharp separation that these genes reveal between different subregions of the UMAP. Placing these UMAPs alongside Figure 3E, where we use genetic reagents to map the expression of *nub* and *TfAP-2* within the leg itself, is the critical demonstration of how we have arrived at our annotations. Figure 3A and 3C further show that these sharp gene expression divides extend beyond the two genes presented in Figure 3B and D. We choose to illustrate *drm* and *bab2* because of their known role in different aspects of leg development: *drm* as being involved in joint formation and *bab2* as being restricted to the tarsus starting with the distal first tarsal segment (Godt *et al.,* 1993), which allows us to distinguish between different regions of the tibia/tarsus joint.

We also believe that these plots are not redundant with respect to each other. The joint UMAP is essential here for comparison with the later joint UMAPs in the figure. But by providing the full epithelial UMAPs alongside, we are demonstrating the degree of joint specificity in these genes. It is through this 3A vs 3C comparison, for example, that you can see that while *drm* is tightly restricted to a subset of joint cells, *bab2* is restricted to one joint population but also more widely expressed among epithelial cells.

To make our intentions for showing both the overall and joint-specific epithelial UMAPs clearer we have now added the following sentence to the Figure 3A-D legend: ‘*bab* is known to be restricted to the tarsus (Godt *et al.*, 1993; Bourbon *et al.*, 2022), providing one means through which to distinguish between the tibia and intertarsal joints.’

4. For Fig. 3E, the TfAP-2 and nub cluster can be further divided into two sub-cluster. It will be interesting to visualize these two gene expressions together in the same fly to further characterize distal and proximal tibia/ta1 joint cells, (GAL4>nlsRFP, tagged-GFP).

As the reviewer notes, the *TfAP-2* and *nub* cluster can be reasonably separated into two subclusters, which we do in Figure 3F after validating that separation with the TfAP-2::GFP and *nub-GAL4* data presented in Figure 3E. We thank the reviewer for the suggested experiment. However, both in the paper as a whole and in our revisions we wanted to focus on sensory organs as much as possible and were reluctant to add even more data on other cell types. Particularly given that the separate stainings themselves provide support for the cluster-specific expression patterns of *TfAP-2* and *nub* that we observe in our single-cell data.

5. Current Fig. 4A and 5A are identical. I suggest the authors highlight cell types that are discussed in Fig. 4 and Fig. 5 accordingly.

We thank the reviewer for this good suggestion. We have now changed the font colours in Fig. 4 and Fig. 5 to highlight the cell populations discussed in the focal figure. We have amended the figure legends accordingly. The legend to Figure 4A now ends with: ‘Cell labels provided in purple indicate populations that are discussed in this figure. Those in black are discussed in Figure 5.’ The legend to Figure 5A now ends with: ‘Cell labels provided in purple indicate populations that are discussed in this figure. Those in black are discussed in Figure 4.’

We recognize that the same UMAP is used at the start of both figures. Our rationale was that reincluding this UMAP in Fig. 5 would provide a quick, labelled guide that the reader could use to interpret the subsequent panels in the figure without having to refer back a previous figure.

6. The authors should provide the general neuronal markers, (elav, nSyb, para) for Fig. 6B.

We thank the reviewer for this suggestion. We have now modified both the 6B and 6H dotplots accordingly. The accompanying figure legend now reads: ‘The dotted lines separate the neuronal markers from the transcription factors and then the chemoreceptor from mechanoreceptor organ transcription factor markers.’

7. Line 395-396, I don't agree with the statement of "…they integrated poorly". The integration pattern between cells from developmental stages seems very normal to me. From development to adult, there are dramatic transcriptomic changes, even for the same cell types. I consider current pattern in Fig. S6 -1I,J as they integrated well (see Xie et al 2021 eLife; McLaughlin et al 2021 eLife)

We understand where the reviewer is coming from here and it is probably fair to describe our use of ‘poorly’ as somewhat of an overstatement. However, it remains true that they did not integrate nearly as well as our 24 and 30 hour datasets, which was our standard for optimal integration, and we stand by our decision to analyze the Fly Cell Atlas (FCA) and our own data separately, particularly in light of the significant differences across multiple variables between our dataset and the FCA data: while the FCA data was single-nuclei ours was single-cell; the FCA data was from adults, ours was from pupae; the FCA data used all leg segments, ours used only the first tarsal segment; the FCA data used all 3 pairs of legs, we used only the forelegs. Finally, the FCA data and our own were generated using dissociation protocols that differed in the chemical composition of the dissociation buffer and in the conditions the dissociating tissues were subjected to, namely whether filtration or centrifugation was used, or mechanical dissociation was performed using a dounce homogenizer or pipette.

Alongside these potential sources of variation, it is our opinion that the separate analysis we conduct – with side-by-side UMAPs – makes for a much clearer demonstration of the conserved and divergent elements of a given cell type’s transcriptome in the pupal and adult datasets.

We have amended the language the reviewer refers to, which now reads as: ‘The pupal and FCA neuron datasets did not integrate as well as our pupal datasets did to each other (S9I and S9J Fig). In light of the significant differences in sample preparation, age, cells vs. nuclei, and dissected region between the datasets we opted to analyze them separately.’

8. Fig. 10 should be in the supplementary Figure.

This point was also raised by Reviewer 1. We agree and have now moved this figure to the supplementary accordingly.

9. There are very limited comparisons between 24h and 30h datasets. It would be great if the authors can compare these two datasets and identify some stage-specific changes. Otherwise, it is not clear why the authors choose these two time points for the experiments.

We share the Reviewer’s interest in between timepoint comparisons. As it stands, we’ve used these sparingly to make points about heterochrony (e.g. in relation to sheath cells, S16 Fig) where we see e.g. (1) a clear expansion of genes that are expressed in a relatively small number of cells in the 24h dataset but are much more widely expressed at 30h and (2) widespread expression across both timepoints in the homologous cell type in a different organ (chemosensory sheaths vs mechanosensory sheaths). To make robust quantitative comparisons between timepoints we would want a degree of per-timepoint replication and, arguably, some additional timepoints to make stronger inferences about change through time. We believe that these analyses are beyond the scope of this already comprehensive and lengthy paper, but it’s something that we are currently generating new data for and hope to publish on separately in the future.

As for why the 24h and 30h timepoints were selected. The reasons were twofold:

(1) 24h is known to fall soon after the sensory organ precursor cell descendants have undergone their final division to form the mature shaft, socket, sheath, and neuron cells of a bristle. To make the importance of this clearer, we have modified the opening line of the final paragraph of the introduction. It now reads: ‘Here, we use scRNA-seq to profile the sensory organs of the male *D. melanogaster* foreleg at two developmental timepoints that follow soon after the specification of sensory organ cells (24h and 30h after puparium formation, APF)(Tanaka *et al.,* 2009)’.

(2) It falls within the developmental window where we can bypass the considerable difficulties of isolating cells from the cuticle. The new lines that we’ve added to the introduction in response to Reviewer 1’s comment about the innovations present in our sample preparation protocol should hopefully now explain the choice of these timepoints.

References:

del Álamo, D., Terriente, J. & Díaz-Benjumea, F.J. (2002) Spitz/EGFr signalling via the Ras/MAPK pathway mediates the induction of bract cells in Drosophila legs. *Development* **129**, 1975–1982. The Company of Biologists.

Bourbon, H.M.G., Benetah, M.H., Guillou, E., Mojica-Vazquez, L.H., Baanannou, A., Bernat-Fabre, S., Loubiere, V., Bantignies, F., Cavalli, G. & Boube, M. (2022) A shared ancient enhancer element differentially regulates the bric-a-brac tandem gene duplicates in the developing Drosophila leg. *PLoS Genetics* **18**, e1010083. Public Library of Science.

Cripps, R.M., Black, B.L., Zhao, B., Lien, C.L., Schulz, R.A. & Olson, E.N. (1998) The myogenic regulatory gene Mef2 is a direct target for transcriptional activation by Twist during Drosophila myogenesis. *Genes and Development* **12**, 422–434. Genes Dev.

Fernandes, J.J., Celniker, S.E. & VijayRaghavan, K. (1996) Development of the indirect flight muscle attachment sites in Drosophila: Role of the PS integrins and the stripe gene. *Developmental Biology* **176**, 166–184. Dev Biol.

Frommer, G., Vorbrüggen, G., Pasca, G., Jäckle, H. & Volk, T. (1996) Epidermal egr-like zinc finger protein of Drosophila participates in myotube guidance. *The EMBO Journal* **15**, 1642–1649. John Wiley & Sons, Ltd.

Godt, D., Couderc, J.L., Cramton, S.E. & Laski, F.A. (1993) Pattern formation in the limbs of Drosophila: Bric a brac is expressed in both a gradient and a wave-like pattern and is required for specification and proper segmentation of the tarsus. *Development* **119**, 799–812. Development.

Held, L.I. (1990) Sensitive periods for abnormal patterning on a leg segment in Drosophila melanogaster. *Rouxs Archives of Developmental Biology* **199**, 31–47. Springer.

Li, H., Watson, A., Olechwier, A., Anaya, M., Sorooshyari, S.K., Harnett, D.P., Lee, H.-K. (Peter), Vielmetter, J., Fares, M.A., Garcia, K.C., Özkan, E., Labrador, J.-P. & Zinn, K. (2017) Deconstruction of the beaten Path-Sidestep interaction network provides insights into neuromuscular system development. *eLife* **6**. eLife Sciences Publications Ltd.

Luecke, D., Rice, G. & Kopp, A. (2022) Sex-specific evolution of a Drosophila sensory system via interacting cis- and trans-regulatory changes. *Evolution and Development* **24**, 37–60. John Wiley and Sons Inc.

Mou, X., Duncan, D.M., Baehrecke, E.H. & Duncan, I. (2012) Control of target gene specificity during metamorphosis by the steroid response gene E93. *Proceedings of the National Academy of Sciences of the United States of America* **109**, 2949–2954. National Academy of Sciences.

Natori, K., Tajiri, R., Furukawa, S. & Kojima, T. (2012) Progressive tarsal patterning in the Drosophila by temporally dynamic regulation of transcription factor genes. *Developmental Biology* **361**, 450–462. Academic Press.

Peng, Y., Han, C. & Axelrod, J.D. (2012) Planar Polarized Protrusions Break the Symmetry of EGFR Signaling during Drosophila Bract Cell Fate Induction. *Developmental Cell* **23**, 507–518.

Reed, C.T., Murphy, C. & Fristrom, D. (1975) The ultrastructure of the differentiating pupal leg of Drosophila melanogaster. *Wilhelm Roux’s Archives of Developmental Biology* **178**, 285–302. Springer-Verlag.

Rodriguez, I., Hernandez, R., Modolell, J. & Ruiz-Gomez, M. (1990) Competence to develop sensory organs is temporally and spatially regulated in Drosophila epidermal primordia. *EMBO Journal* **9**, 3583–3592.

Sasse, S. & Klämbt, C. (2016) Repulsive Epithelial Cues Direct Glial Migration along the Nerve. *Developmental Cell* **39**, 696–707. Cell Press.

Simon, F., Fichelson, P., Gho, M. & Audibert, A. (2009) Notch and Prospero repress proliferation following cyclin E overexpression in the Drosophila bristle lineage. *PLoS Genetics* **5**, e1000594. Public Library of Science.

Tanaka, K., Barmina, O. & Kopp, A. (2009) Distinct developmental mechanisms underlie the evolutionary diversification of Drosophila sex combs. *Proceedings of the National Academy of Sciences* **106**, 4764–4769.

Volohonsky, G., Edenfeld, G., Klämbt, C. & Volk, T. (2007) Muscle-dependent maturation of tendon cells is induced by post-transcriptional regulation of StripeA. *Development* **134**, 347–356. Development.

Wayburn, B. & Volk, T. (2009) LRT, a tendon-specific leucine-rich repeat protein, promotes muscle-tendon targeting through its interaction with Robo. *Development* **136**, 3607–3615. The Company of Biologists.
